# Fungal Periprosthetic Joint Infection: A Systematic Review and Meta-analysis of Treatment Approaches and Clinical Outcomes

**DOI:** 10.1093/ofid/ofag657

**Published:** 2026-10-08

**Authors:** Farzad Pourghazi, Seyed Mohammad Amin Alavi, Takahiro Matsuo, Georges Baaklini, Matthew P Abdel, Aaron J Tande, Elie F Berbari

**Affiliations:** Division of Public Health, Infectious Diseases and Occupational Medicine, Department of Medicine, Mayo Clinic College of Medicine and Science, Mayo Clinic, Rochester, Minnesota, USA; Faculty of Medicine, Ahvaz Jundishapur University of Medical Sciences, Ahvaz, Iran; Department of Infectious Diseases, Infection Control, and Employee Health, The University of Texas MD Anderson Cancer Center, Houston, Texas, USA; Faculty of Medicine, Saint Joseph University, Beirut, Lebanon; Department of Orthopedic Surgery, Mayo Clinic, Rochester, Minnesota, USA; Division of Public Health, Infectious Diseases and Occupational Medicine, Department of Medicine, Mayo Clinic College of Medicine and Science, Mayo Clinic, Rochester, Minnesota, USA; Division of Public Health, Infectious Diseases and Occupational Medicine, Department of Medicine, Mayo Clinic College of Medicine and Science, Mayo Clinic, Rochester, Minnesota, USA

**Keywords:** exchange arthroplasty, fungal prosthetic joint infection, meta-analysis, outcome

## Abstract

Fungal periprosthetic joint infection (PJI) is a rare but serious complication of joint arthroplasty, with variable management and poorly defined outcomes. We conducted a systematic review and meta-analysis in accordance with PRISMA guidelines, searching Ovid MEDLINE, Embase, Scopus, and Ovid Cochrane Central. Cohort studies were included in the quantitative synthesis, while case reports and series were reviewed qualitatively. Outcomes were treatment success, failure, reinfection, and mortality. We included 160 studies: 42 cohort studies and 118 case reports or case series, with 1025 fungal PJI cases. Pooled rates were 66.5% for treatment success, 36.5% for failure, 16.6% for reinfection, and 9.6% for mortality. Success and failure were extracted independently using study-specific definitions and are not necessarily complementary. Treatment-strategy estimates were descriptive; studies reporting DAIR had lower pooled success, but findings should be interpreted cautiously because of differences in patient selection, study design, and outcome definitions. These findings cannot establish comparative effectiveness.

## Teaser

Fungal periprosthetic joint infection is a rare but serious arthroplasty complication. In this systematic review and meta-analysis of 160 studies and 1025 cases, pooled treatment success was 66.5%, while failure, reinfection, and mortality remained substantial. Pooled outcomes varied across surgical strategies, with less favorable results reported in studies including DAIR; these findings are descriptive and should be interpreted cautiously.

Total joint arthroplasty (TJA) is among the most performed orthopedic procedures worldwide. In the United States, the annual number of primary total knee arthroplasty (TKA) and total hip arthroplasty (THA) procedures is projected to increase substantially, reaching approximately 3.48 million TKA procedures and 572 000 THA procedures by 2030 [1]. Periprosthetic joint infection (PJI) is a relatively uncommon yet clinically distinct complication that differs in presentation and pathophysiology from infections of native bones or joints. It occurs in approximately 2% of patients undergoing arthroplasty and has a subtle epidemiology that evolves over time [2]. Periprosthetic joint infection is mostly caused by bacterial pathogens; however, in rare cases, fungal organisms may be responsible, accounting for nearly 1% of all PJIs [2–4]. As the number of TJAs continues to grow, the total number of fungal PJIs is anticipated to increase. The most reported fungal pathogen in the literature is *Candida albicans*, followed by *Candida parapsilosis* and *Candida glabrata* [5].

Several potential risk factors for fungal PJI have been identified in the literature, including immunosuppression, diabetes mellitus, prior or inappropriate antimicrobial use, prior revision surgeries, and wound drainage [6, 7]. It should be noted that systemic inflammatory markers and synovial fluid cell count analyses are unable to differentiate between fungal and bacterial PJI [8].

The ability of fungal organisms to adhere to prosthetic surfaces, form biofilms, and exhibit resistance to antifungal agents poses significant challenges to management [9]. Various treatment protocols have been described in the literature, including debridement, antibiotics, and implant retention (DAIR), 1-stage exchange arthroplasty, 2-stage exchange arthroplasty, resection arthroplasty, and amputation. Among these, the 2-stage exchange protocol has been the most commonly employed approach [10].

Although several studies have reported management approaches for fungal PJI, data on treatment outcomes remain limited and inconsistent, and no quantitative synthesis has been available. We conducted a systematic review and meta-analysis designed to establish benchmark outcome proportions for fungal PJI across surgical strategies.

## MATERIALS AND METHODS

### Study Design and Search Strategy

The present systematic review and meta-analysis were conducted and reported in accordance with the updated Preferred Reporting Items for Systematic Reviews and Meta-Analyses (PRISMA) guidelines [11]. A comprehensive search of several databases was performed on November 18, 2025. Results were limited to the English language; no date limits were applied. Animal studies were excluded. Ovid MEDLINE, Ovid Embase, Ovid Cochrane Central Register of Controlled Trials, and Scopus were searched.

The search strategies were designed and conducted by a medical librarian with input from the study investigators. Controlled vocabulary supplemented with keywords was used. The actual strategies listing all search terms used and how they are combined are presented in the Supplementary Data.

### Protocol

A predefined protocol was developed and internally approved prior to study initiation; however, it was not prospectively registered in a publicly accessible registry.

### Study Selection

Title, abstract, and full-text screening were conducted in COVIDence (Veritas Health Innovation, Melbourne, Australia). Two reviewers (F. P. and S. M. A. A.) independently screened all records, and disagreements were resolved by discussion with third reviewer input (T. M.) when needed. We included English-language studies of adults (≥18 years) with fungal PJI, regardless of diagnostic criteria, which reported medical and surgical management and outcomes. Because fungal PJI is rare, case reports are also included. We excluded pediatric studies and review articles, systematic reviews, meta-analyses, study protocols, and preprints. The primary objective was to evaluate outcomes of management strategies for fungal PJI.

### Data Extraction

A data extraction form was initially piloted in Excel using a sample of ten studies, with 2 reviewers (F. P. and S. M. A. A.) participating in this phase. Based on feedback and recommendations from the research team, the form was refined iteratively to minimize misunderstandings and resolve potential discrepancies. Subsequently, 3 reviewers (F. P., S. M. A. A., G. B.) extracted relevant data from all included studies, recording the information in Excel spreadsheets.

Treatment strategies were extracted as reported in each included study. When studies reported more than one intervention for the same patient over the course of treatment, such as DAIR followed by exchange arthroplasty after treatment failure, each reported procedure was extracted and classified according to the corresponding treatment strategy. Therefore, treatment strategy classification was based on reported procedures rather than strictly on mutually exclusive patient-level groups. As a result, some patients may have contributed to more than one treatment subgroup when sequential interventions were reported. Accordingly, treatment-strategy groups were not mutually exclusive at the patient level, and treatment-specific denominators are reported as outcome observations that may represent patients, joints, or procedures, depending on the source study. Outcome data were extracted according to definitions reported by each study and are described in detail below.

### Outcome Definition

Because outcome definitions varied across included studies, outcomes were extracted and reported according to each study's original definitions whenever available. When definitions were not explicitly provided, outcomes were classified based on the authors’ descriptions of infection status, need for additional surgery, microbiologic recurrence, or death. Because success, failure, and reinfection were not consistently defined as complementary outcomes across studies, each outcome was analyzed independently.

### Risk of Bias and Quality of Evidence Assessment

All included cohort studies were assessed for quality and risk of bias using the Newcastle–Ottawa Scale (NOS) [12]. Because most included studies were retrospective, non-comparative cohorts without a control group, the non-exposed cohort and comparability domains were frequently not applicable. For these studies, quality assessment focused primarily on the applicable selection and outcome domains. Comparability was evaluated only when an explicit comparison group was present and adjustment or matching for potential confounders was reported. Adequate follow-up was defined a priori as ≥6 months. The NOS was selected because it is commonly used for observational cohort studies; however, its application to non-comparative cohorts has recognized limitations, including variability in scoring and limited assessment of confounding. Therefore, NOS results were used as descriptive indicators of methodological quality rather than definitive measures of risk of bias. Detailed domain-level assessments are provided in Supplementary Table 3.

Case reports and case series were assessed using the Murad et al tool [13]. Quality assessment was used to inform interpretation, not study exclusion. Detailed assessments are provided in Supplementary Table 4. Quality assessment findings were used to inform interpretation of the results rather than to exclude studies or to perform quality-based sensitivity analyses.

### Statistical Analysis

All analyses were performed in R 4.4.2 with RStudio. Because clinical and methodological heterogeneity was expected, pooled estimates were calculated using random-effects models. Only cohort studies with at least 5 fungal PJI patients were included in the meta-analysis. Studies included in the quantitative meta-analyses are indicated in Table 1, while cohorts with at least 5 fungal PJI patients were eligible for quantitative synthesis. For treatment-strategy subgroup analyses, a treatment subgroup was included only when at least 5 evaluable observations were available for that strategy. Outcome proportions were pooled using random-intercept logistic regression with logit transformation and maximum-likelihood estimation of τ^2^ and are reported with 95% confidence intervals (CIs). This approach was selected to account for variability in study sizes and to provide stable estimates of proportions, particularly for outcomes with extreme values. Heterogeneity was assessed using Cochran's Q test and the I^2^ statistic. Meta-regression with publication year and follow-up duration was performed to explore heterogeneity. When heterogeneity was substantial, leave-one-out sensitivity analysis was used. Subgroup analyses were limited to 1-stage exchange, 2-stage exchange, and DAIR because other interventions had insufficient data. Because treatment-strategy groups were not mutually exclusive and individual patients could contribute to more than one procedural category when sequential interventions were reported, treatment-strategy subgroup analyses were considered exploratory and descriptive. Subgroup estimates were not interpreted as comparative effectiveness estimates. Formal tests of differences between treatment-strategy subgroups were not performed because the independence assumption could not be assured. Publication bias was assessed by funnel plot inspection and Egger's regression test when at least 10 studies were available. Given the limited number of studies for some outcomes, trim-and-fill analyses were not performed.

**Table 1.** General Characteristics and Findings of Cohort Studies

Author, Year	Country	N	Joint	Age (Mean or Range)	Definition for DIAGNOSIS of PJI Used by the Authors	Follow-up (Mean)	Organism	Treatment	Outcome
Anagnostakos, 2021 [14]	Germany	3	Hip: 1 Knee: 2	NR	MSIS 2011	13–24	*C albicans*: 2, *C guilliermondii*: 1	DAIR and two-stage exchange	One failure, one death, one not extractable
Anagnostakos, 2012 [15]	Germany	7	Hip: 4 Knee: 3	68	Culture-based	28	*C albicans*: 3, *C glabrata*: 2, *C lipolytica*: 1, *C parapsilosis*: 1, *C curvatum*: 1 (one patient with two organism)	Two-stage exchange	All successful (7/7)
Azzam, 2009 [16]	USA	31	Hip:14 Knee:17	64	Culture-based	45	*C albicans*: 23, *C glabrata*: 1, *C parapsilosis*: 7, *Aspergillus* spp.: 1, *Rhodotorula minuta*: 1, *Aureobasidium*: 1 (3 patient with both *C albicans* + *C parapsilosis*)	DAIR and two-stage exchange and resection + spacer	9/26 success, failure 17/26 (including one death 1/26)
Baecker, 2021 [17]	Germany	18	Hip: 11 Knee: 7	72.8	EBJIS 2021	35	*C albicans*: 7, *C glabrata*: 1, *C parapsilosis*: 5, *C famata*: 3, *C tropicalis*: 1, *Alternaria infectoria*: 1	Three-stage exchange	16/18 success, 2/18 failure (including one death)
Brown, 2018 [18]	USA	31	Hip: 13 Knee: 18	37–92	MSIS 2011	48	*C albicans* 15, *C parapsilosis* 7, *C glabrata* 1, *C tropicalis* 1, *C guilliermondii* 1, *Alternaria* spp. 2, *Aspergillus versicolor* 1, *Coccidioides immitis* 1, *Aureobasidium* and *Hormonema* spp. 1, *Phitomyces* spp. 1	Two-stage, DAIR, one-stage exchange retention, resection arthroplasty, antifungal suppression, spacer retained in situ	16/31 success, 15/31 failure (including 12/31 reinfections), two-stage: 7 success, 7 failure. One-stage exchange: 1 success, DAIR: 4 failures, retained spacers: 1 success and 1 failure. Treatment-specific outcome was not reported for the remaining 10 patients
Burr, 2022 [19]	USA	1	Hip: 1	NR	None	NR	*C albicans*: 1	Nonoperative treatment	Successful treatment
Dinh, 2025 [5]	Multi-national	269	Hip: 146 Knee: 118 Shoulder: 4 Ankle: 1	73	EBJIS 2021	≥24	*C albicans*: 150; *C glabrata*: 21; *C parapsilosis*: 79; *C tropicalis*: 15; *C dubliniensis*: 3; *C metapsilosis*: 4; *C orthopsilosis*: 2; *C krusei*: 2; *C kefyr*: 1; *C lusitaniae*: 1 (some had combined infection, combinations, and number of patients with combination NR)	DAIR: 96 One-stage exchange: 76 Two-stage exchange: 78 Resection arthroplasty: 7 Salvage: 4 Amputation: 2 (strategy for 6 patients NR)	Overall: success: 156/269, failure 113/269, 52/269 reinfection; DAIR: 96 (45 success, 51 failure); one-stage exchange: 76 (51 success, 25 failure); 2-stage exchange: 78 (54 success, 24 failure); definitive resection: 7 (3 success, 4 failure); salvage: 4 (2 success, 2 failure) and amputations (1 success and 1 failure), 6/269 treatment-based outcome NR
Eckers, 2021 [20]	Switzerland	1	Knee: 1	49	None	NR	*C albicans*: 1	DAIR and then amputation	Failure
Enz, 2021 [21]	Germany	13	NR	76.5	Culture-based	NR	*C albicans*: 16; *C glabrata*: 1; *C parapsilosis*: 1 (some had repeated culture tests for *Candida*)	Two-stage and DAIR and resection	10/13 success, 3/13 failure
Escola-Verge, 2018 [22]	Spain	43	Hip: 26 Knee: 16 Shoulder: 1	75	Culture-based	≥24	*C albicans*: 24; *C parapsilosis*: 14; *C glabrata*: 2; *C tropicalis*: 1; mixed *Candida* spp.: 2	15/43 DAIR; 28/43 mixed	Outcome reported for 35/43, overall: 17/35 success, 18/35 failure; DAIR: 15 (4 success, 11 failure); prosthesis removal: 20 (13 success, 7 failure)
Gao, 2018 [23]	China	17	Hip: 5 Knee: 13 (17 patients but 18 joints)	61.2	Culture-based diagnosis	65	*C parapsilosis*: 6; *C albicans*: 5; *C glabrata*: 2; *C tropicalis*: 2; *C freyschussii*: 1; *Aspergillus* spp.: 2; *A strictum*: 1 (some had multiple organisms)	Two-stage exchange and DAIR	13/23 success, 10/23 failure Two-stage revision: 18 (13 success, 5 failure) DAIR (5 failures and converted to two-stage)
Geng, 2016 [24]	China	8	Hip: 4 Knee: 4	60	Culture-based diagnosis	≥24	*C albicans*: 3; *C glabrata*: 1; *C parapsilosis*: 1; *C freyschussii*: 1; *Aspergillus* spp.: 1; other mold: 1	Two-stage exchange:7 Resection arthroplasty: 1	Two-stage exchange: 7 (6 success, 1 failure); resection arthroplasty: 1 (success)
Gonzalez, 2024 [25]	USA	10	Knee: 10	67	MSIS 2011	26	*C albicans*: 5; *C parapsilosis*: 3; *C tropicalis*: 1; *Nakaseomyces (Candida) glabrata*: 1	DAIR and two-stage exchange	1/10 success, 9/10 failure including 1/10 death
Hartman, 2022 [26]	USA	2	NR	NR	Culture-based	≥24	NR	Two-stage exchange	2/2 success
Herndon, 2023 [27]	USA	41	Hip: 22 Knee: 19	64	MSIS 2011	≥12	*Candida* spp.: 39; non-*Candida* fungi: 2	Two-stage exchange and DAIR	20/41 success, 21/41 failure
Hwang, 2012 [28]	Korea	28	Knee: 30 (two bilateral)	69	Culture-based	≥24	*C parapsilosis*: 12; *C albicans*: 12; other *Candida* spp. (including *Pichia anomala*): 6	Two-stage exchange	28/30 success, 2/30 reinfection
Jeon, 2023 [29]	Korea	1	Knee: 1	71	MSIS 2014	13–24	*C albicans*: 1	DAIR: 1	Successful treatment
Ji, 2022 [30]	China	7	NR	NR	MSIS 2011	≥24	NR	One-stage exchange	4/7 success, 3/7 failure
Ji, 2019 [31]	China	5	Hip: 5	NR	MSIS 2011	≥24	*C albicans*: 1, *C glabrata*: 2, *C parapsilosis*: 1, *Aspergillus* spp.: 1	One-stage exchange	4/5 success, 1/5 relapse
Ji, 2017 [32]	China	11	Hip: 4 Knee: 7	66	MSIS 2011	60	*C albicans*: 6; *C parapsilosis*: 2; *C glabrata*: 2, *C pseudotropicalis*: 1	One-stage exchange	7/11 success, 3/11 reinfection, 1/11 death
Kanar, 2023 [33]	Turkey	1	Knee: 1	74	MSIS 2011	13–24	*C albicans*: 1	Arthrodesis 1	Success
Karczewski, 2022 [34]	Germany	29	Hip: 14 Knee: 15	71	NR	≥24	*C albicans*: 17; *C parapsilosis*: 10; *C glabrata*: 1; *C tropicalis*: 1; *C guilliermondii*: 1 (one patient with combined infection)	Mixed procedures	Overall: 8/29 reinfection, 21/29 not extractable
Kazimoglu, 2015 [35]	Turkey	1	Hip: 1	NR	Culture-based	7–12	*C albicans*: 1	DAIR: 1	Successful treatment
Kim, 2018 [36]	Korea	9	Knee: 9	76	MSIS 2011	66	*C parapsilosis*: 8; *C pelliculosa*: 1	Two-stage exchange	All successful 9/9
Kim, 2021 [37]	Korea	6	Knee: 6	67	MSIS 2011	≥24	*C albicans*: 3, *C lusitaniae*: 3	Two-stage exchange	2/6 success, 4/6 failure
Klatte, 2014 [38]	Germany	10	Hip: 6 Knee: 4	68	MSIS 2011 criteria	84	*C albicans*: 5; *C glabrata*: 1; *C parapsilosis*: 3; *C lusitaniae*: 1	One-stage exchange	9/10 success, 1/10 failure
Kuo, 2018 [39]	USA	29	Hip: 14 Knee: 15	70.7 ± 8.7	MSIS 2011	≥24	*C albicans*: 7; *C parapsilosis*: 6; *C tropicalis*: 4; *C glabrata*: 1; *Aspergillus* spp.: 3; *Penicillium* spp.: 2; *Rhodotorula minuta*: 2; *Blastoschizomyces capitatus*: 1; *Verticillium* spp.: 1 (two organisms were NR)	DAIR and one-stage exchange and two-stage exchange arthroplasty	14/29 success, 15/29 failure (including 2/29 deaths)
Lachman, 2018 [40]	USA	1	Ankle: 1	66	Other	≥24	*C albicans*: 1	DAIR: 1	Failure
Lee, 2024 [41]	South Korea	12	Knee: 12	NR	MSIS 2018	≥36	NR	Two-stage exchange	5/12 success, 7/12 failure
McCulloch, 2023 [42]	United Kingdom	69	Hip: 24 Knee: 45	68	MSIS 2011	34	*C albicans* 28, *C parapsilosis* 17, *C glabrata* 5, *C dubliniensis* 4, *C tropicalis* 3, *Aspergillus fumigatus* 4, *Candida species* 8	Mixed procedures	33/69 success, 36/69 failure
Miller, 2015 [43]	USA	8	Hip: 5 Knee: 2 Shoulder: 1	Range 22–83	Other	17	NR	Surgical debridement + removal of prosthesis	8/8 success
Phelan, 2002 [44]	USA	10	Hip: 6 Knee: 4	Range 37–83.2	Other	50.7	*C albicans* 6, *C parapsilosis* 3, *C tropicalis* 1	Two-stage exchange	8/10 success, 2/10 reinfection
Saconi, 2020 [45]	Brazil	11	Hip: 6 Knee: 5	Range 52–85	Other	NR	*C albicans* 6, *C parapsilosis* 4, *C glabrata* 1, *C lusitaniae* 1, *Aspergillus* 1 (some had combined organism)	DAIR and one-stage exchange and two-stage exchange and resection arthroplasty	Overall: 8/9 success, 1/9 failure, 2/11 NR, 1/9 reinfection, 2/11 NR DAIR: 1 success, 0 failure One-stage exchange: 2 success, 1 failure, 1 NR Two-stage exchange: 1 success, 0 failure Resection arthroplasty: 4 success, 1 NR
Sidhu, 2019 [46]	United Kingdom	22	Hip: 8 Knee: 14	64.5	MSIS 2018	49	*C albicans* 9, *C parapsilosis* 8, *Candida* species mixed 1, *A fumigatus* 4	Two-stage exchange	9/22 success, 13/22 failure
Starnes, 2024 [47]	USA	18	Hip: 9 Knee: 9	61.9	Other	≥12	*C albicans* 12, *C parapsilosis* 4, *C glabrata* 1, *C dubliniensis* 1, *T inkin* 1 (one patient had mixed fungal infection)	DAIR and one-stage exchange and two-stage exchange, Girdlestone procedure, amputation	Outcome was available for 11/18 patients; 7/18 had no reported outcome. Among evaluable patients, 4/11 were successful and 7/11 failed. Two-stage exchange: 3/7 success, 4/7 failure
Theil, 2019 [48]	Germany	26	Hip: 18 Knee: 8	72	MSIS 2011 criteria	33 m	*C albicans* 19, *C parapsilosis* 3, *C glabrata* 2, *C dubliniensis* 1, *C famata* 1, *C guilliermondii* 1 (some combined organisms)	Two-stage exchange and one-stage exchange and implant retention + antifungal therapy: 1 failure	Overall: 10/26 success, 16/26 failure. Two-stage exchange: 10 success, 13 failure. One-stage exchange: 0 success, 2 failure Implant retention + antifungal therapy: 1 failure
Ueng, 2013 [49]	Taiwan	16	Hip: 7 Knee: 9	62	NR	41 m	*C albicans* 9, *C parapsilosis* 6, *C tropicalis* 1	Two-stage exchange, resection arthroplasty	8/16 success, 8/16 failure (including 4/16 deaths)
Wang, 2020 [50]	China	1	Knee: 1	NR	NR	38 m	*Candida* species 1	One-stage exchange: 1	Success
Wang, 2015 [51]	China	5	Knee: 5	67	Own definition	41.6 m	*C parapsilosis* 3, *C utilis* 1, *Pichia anomala* 1	Two-stage exchange	5 success
Whiteside, 2017 [52]	USA	1	Hip: 1	NR	MSIS 2011 criteria	≥24 m	*C albicans*: 1	One-stage exchange	Success
Xu, 2024 [53]	China	8	Hip: 3 Knee: 5	67.5	Other	≥24 m	*C guilliermondii* 3, *C parapsilosis* 3, *C pelliculosa* 1, *Aspergillus* 1	Two-stage exchange, knee arthrodesis	6/6 success, 2 knee arthrodesis: 2 success (infection eradicated in all cases)
Yang, 2023 [54]	South Korea	41	Knee: 41	77.6	MSIS 2011 criteria	39 m	*C albicans*: 2, *C glabrata*: 7, *C parapsilosis*: 30, *C pelliculosa*: 1, *C rugosa* 1	46 procedures for 41 patients including two-stage exchange and DAIR	Overall: 26/46 success, 20/46 failure (including 10/46 reinfection), two-stage exchange: 26/36 success, 10/36 failure (reinfection), DAIR: 10 failures

Abbreviations: *A*, *Aspergillus*; *C*, *Candida*; DAIR, debridement, antibiotics, and implant retention; DIAGNOSIS, diagnostic definition/criteria used by study authors; EBJIS, European Bone and Joint Infection Society; f/u, follow-up; fPJI, fungal periprosthetic joint infection; IDSA, Infectious Diseases Society of America; MSIS, Musculoskeletal Infection Society; N, number of patients; NR, not reported; PJI, periprosthetic joint infection; THA, total hip arthroplasty; TKA, total knee arthroplasty; USA, United States of America.

### Patient Consent Statement

This study used data from previously published works only; therefore, patient consent is not applicable.

## RESULTS

### Search Results

The search identified 2492 records. After removing 1014 duplicates, 1478 records were screened, and 206 full texts were sought. Five reports could not be retrieved, leaving 201 full texts assessed for eligibility. Forty-one studies were excluded: 36 did not report outcomes separately for fungal PJI, and 5 did not confirm fungal PJI. Overall, 160 studies were included in the systematic review (Figure 1).

**Figure 1.**
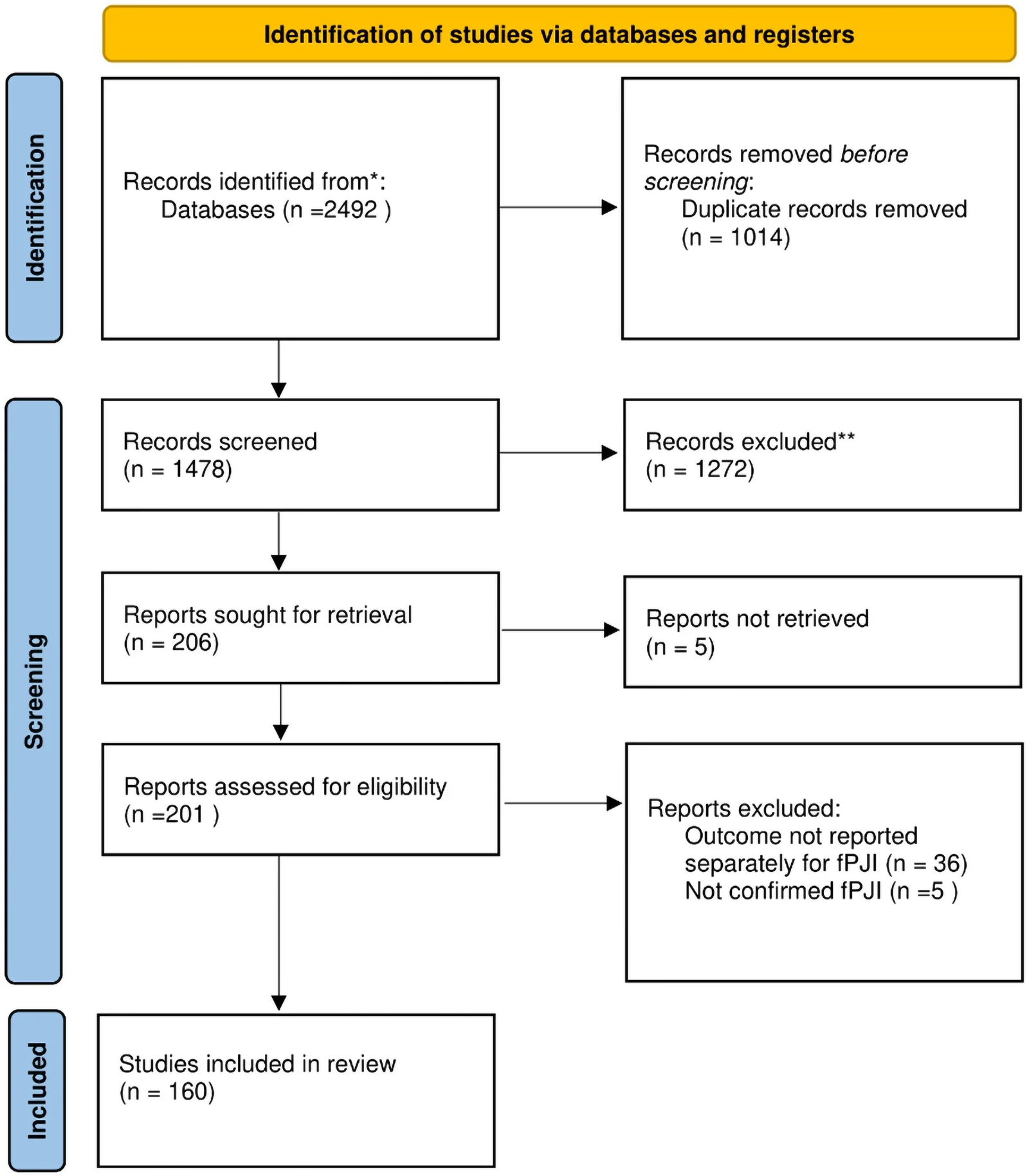
PRISMA flow diagram of study identification, screening, eligibility assessment, and inclusion.

### Study Characteristics

Across 160 included studies, there were 42 cohort studies [5, 14–54] and 118 case reports/series comprising 1025 fungal PJI cases involving mainly THA and TKA, with less frequent shoulder, ankle, elbow, and small-joint involvement (Table 1; Supplementary Tables 1 and 2). Among studies with extractable joint-level data, the knee was most affected (54.8%), followed by the hip (43.2%), shoulder (1.5%), and other joints. Patients were generally older adults, most often in the sixth to eighth decades of life, and fungal PJI usually followed arthroplasty for osteoarthritis, with trauma and malignancy less common as index indications.

Candida species accounted for approximately 93.6% of extractable fungal isolates, most commonly *C albicans* (46.0%) and *C parapsilosis* (29.1%), followed by *C glabrata* (7.4%) and *Candida tropicalis* (4.2%). Non-*Candida* yeasts, filamentous fungi such as *Aspergillus*, mixed fungal infections, and rare molds were reported sporadically. Mixed bacterial–fungal infections were reported in several studies, although the proportion varied and was inconsistently reported across cohorts.

Surgical strategies varied widely and included DAIR, 1-stage, 2-stage, and 3-stage exchange, resection arthroplasty, arthrodesis, amputation, and chronic suppressive antifungal therapy. Two-stage exchange was the most frequently reported approach in cohort studies, whereas case reports and series showed greater heterogeneity. Follow-up ranged widely, from a few months to over 10 years, with isolated cases exceeding 30 years; most studies in the quantitative synthesis had at least 6 months of follow-up. Treatment success was the most frequently reported outcome, while failure, reinfection, and mortality were less consistently reported.

### Meta-analysis Results

Quantitative meta-analyses were restricted to cohort studies reporting extractable outcome data. A total of 31 cohort studies were included in the success meta-analysis [5, 15–18, 21–25, 27, 28, 30–32, 36–39, 41–49, 51, 53, 54], 27 in the failure meta-analysis [5, 15–18, 21–25, 27, 30, 36–39, 41–43, 45–49, 51, 53, 54], 14 in the reinfection meta-analysis [5, 15, 18, 28, 31, 32, 34, 36, 38, 39, 44, 45, 51, 54], and 9 in the mortality meta-analysis [16–18, 25, 32, 39, 47, 49, 51].

Across cohort studies included in the meta-analysis, the pooled analysis included 830 observations for treatment success, 774 for treatment failure, 490 for reinfection, and 157 for mortality. These denominators represent reported outcome observations that may be patient-, joint-, or procedure-level, depending on the source study, and should not be interpreted as counts of unique patients across analyses. One-stage and 2-stage exchange arthroplasty and DAIR were sufficiently represented to allow exploratory descriptive subgroup analyses.

Detailed characteristics of cohort studies, including diagnostic criteria, organisms, surgical strategies, follow-up duration, and outcomes, are summarized in Table 1 and detailed information for outcome based on treatment strategies reported in Supplementary Table 2, while characteristics of case reports and case series are summarized in Supplementary Table 1.

The quantitative synthesis focused on 4 prespecified outcomes: treatment success, treatment failure, reinfection, and mortality. Separate meta-analysis was performed for each outcome to estimate pooled event rates and heterogeneity. Where sufficient data were available, exploratory descriptive subgroup analyses summarized outcomes reported for 1-stage exchange arthroplasty, 2-stage exchange arthroplasty, and DAIR. Because these treatment categories were not necessarily mutually exclusive at the patient level, subgroup estimates were not considered direct comparisons between treatment strategies. Subgroup analysis by joint type was not performed because most studies did not report outcomes separately by joint. Studies with fewer than 5 fungal PJI cases were excluded from the quantitative meta-analysis. Detailed results for each outcome are presented below.

Because treatment success and failure were extracted according to study-specific definitions and were not consistently reported as complementary outcomes across cohorts, their pooled proportions should not be interpreted as summing to 100%. Accordingly, each outcome was meta-analyzed independently.

#### Treatment Success

The success meta-analysis included 31 cohort studies with 830 observations and 481 successes. The pooled success rate was 66.5% (95% CI 55.5–76.0) with moderate heterogeneity (I^2^ = 44.8%, *P* = .0042) (Figure 2). In descriptive subgroup analyses, pooled success estimates were 68.8% for 1-stage exchange, 69.7% for 2-stage exchange, and 13.3% for DAIR. These subgroup-specific pooled estimates are presented for descriptive purposes only and should not be interpreted as formal comparisons of treatment effectiveness because the included studies were non-comparative and treatment strategy groups were not necessarily mutually exclusive. Differences in pooled estimates may also reflect differences in patient selection, clinical characteristics, and study-level factors (Supplementary Figure 1). Egger's test indicated statistically significant funnel plot asymmetry (*P* = .0281) (Supplementary Figure 2). Excluding outlier studies [16, 25, 28] did not materially change the pooled estimate (66.3%, 95% CI 56.1–75.2) and reduced heterogeneity (I^2^ = 20.7%, *P* = .1643). Influence analyses also showed that the pooled estimate remained broadly stable (Supplementary Figure 3).

**Figure 2.**
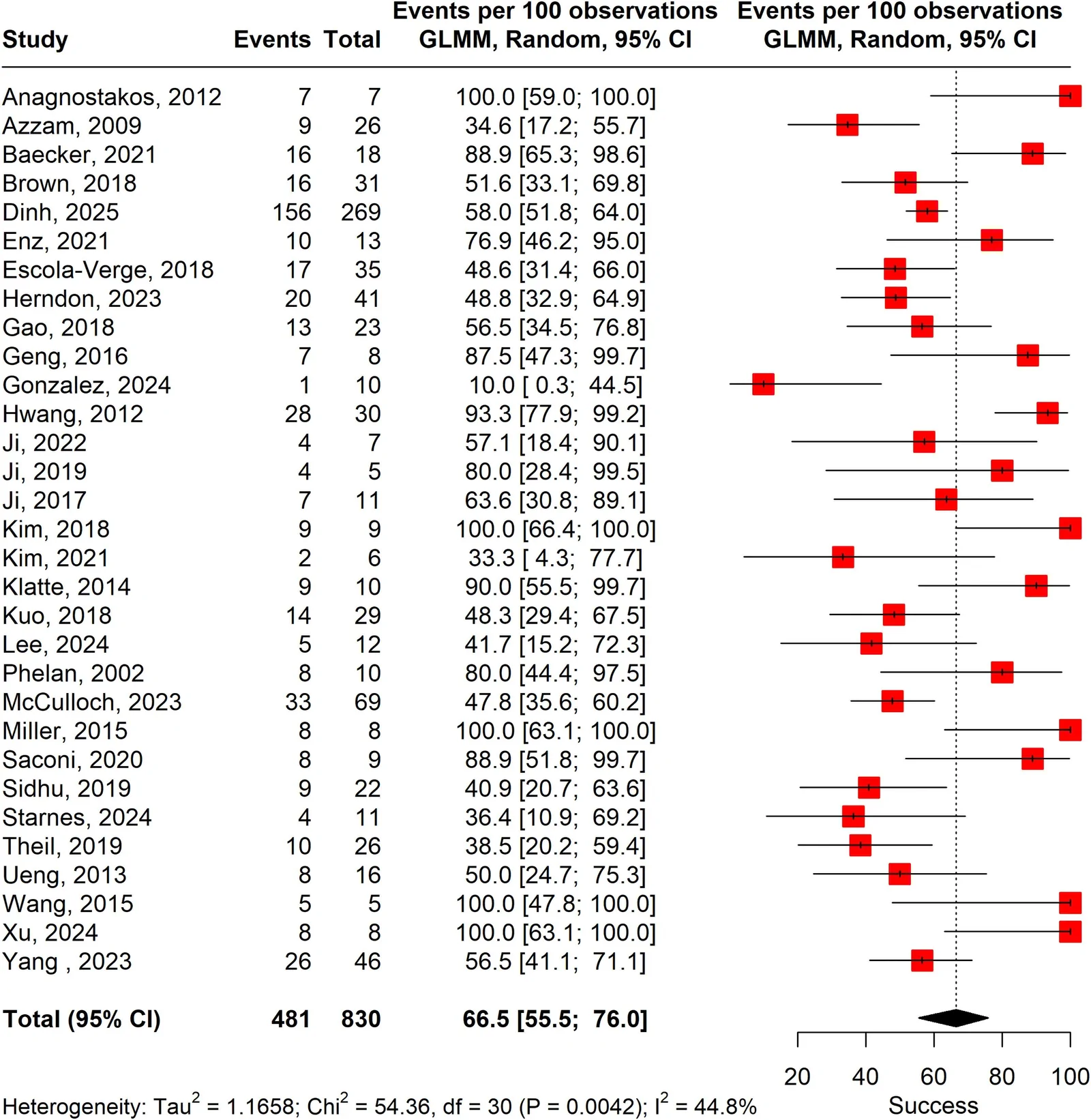
Forest plot of pooled treatment success rates for fungal periprosthetic joint infection (fPJI) using a random-effects generalized linear mixed-effects model (GLMM). The analysis included 31 cohort studies comprising 830 observations and 481 treatment successes. The pooled treatment success rate was 66.5% (95% CI, 55.5–76.0), with moderate heterogeneity (I^2^ = 44.8%).

#### Treatment Failure

The failure meta-analysis included 27 cohort studies with 774 observations and 340 failures. The pooled failure rate was 36.5% (95% CI 26.1–48.3) with moderate heterogeneity (I^2^ = 32.9%, *P* = .0517) (Figure 3). Because treatment success and failure were analyzed independently using study-specific definitions, these pooled proportions should not be interpreted as summing to 100%. In descriptive subgroup analyses, pooled failure estimates were 31.2% for 1-stage exchange, 34.6% for 2-stage exchange, and 86.7% for DAIR. These subgroup-specific pooled estimates are presented for descriptive purposes only and should not be interpreted as formal comparisons of treatment effectiveness because the included studies were non-comparative and treatment strategy groups were not necessarily mutually exclusive. Differences in pooled estimates may also reflect differences in patient selection, clinical characteristics, and study-level factors (Supplementary Figure 4). Egger's test and funnel plot inspection showed no clear asymmetry (Supplementary Figure 5). One random-effects outlier was identified [25]; excluding this study did not materially change the pooled estimate (35.35%, 95% CI 25.6–46.5) and reduced heterogeneity (I^2^ = 26.1%, *P* = .1112). Influence analyses showed that the pooled estimate remained broadly stable (Supplementary Figure 6).

**Figure 3.**
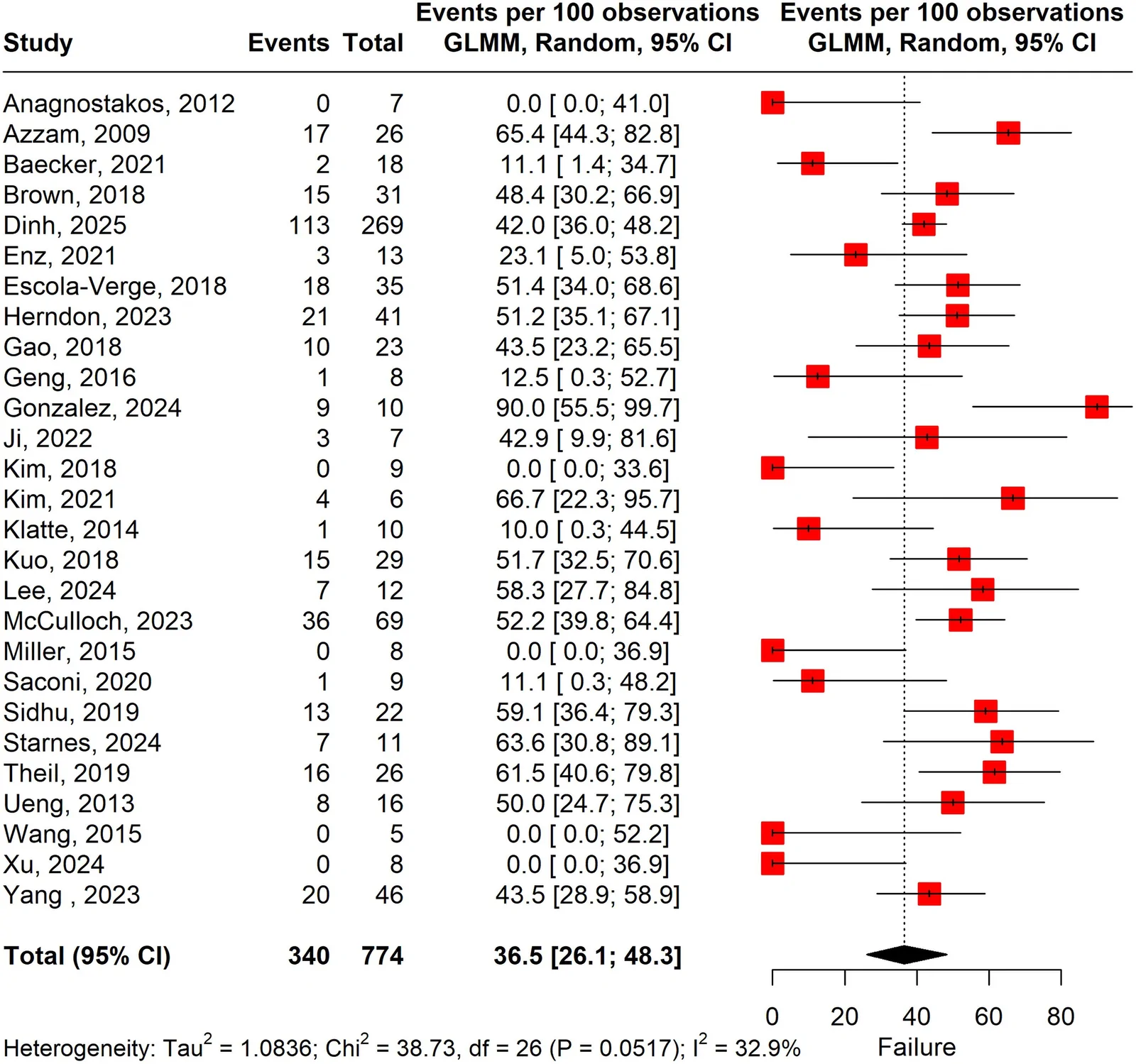
Forest plot of pooled treatment failure rates for fungal periprosthetic joint infection (fPJI) using a random-effects generalized linear mixed-effects model (GLMM). The analysis included 27 cohort studies comprising 774 observations and 340 treatment failures. The pooled treatment failure rate was 36.5% (95% CI, 26.1–48.3), with moderate heterogeneity (I^2^ = 32.9%).

#### Reinfection

The reinfection meta-analysis included 14 cohort studies with 490 observations and 94 reinfections. The pooled reinfection rate was 16.6% (95% CI 10.6–24.9) with low heterogeneity (I^2^ = 15.6%, Q test *P* = .28) (Figure 4). Although overall reinfection included all surgical strategies, subgroup analysis was limited to exchange arthroplasty strategies. Reinfection rates were 19.2% (95% CI 8.2–38.7; I^2^ = 0%) for 1-stage exchange and 12.6% (95% CI 5.2–27.4; I^2^ = 20.3%) for 2-stage exchange. These subgroup estimates are presented descriptively and should not be interpreted as evidence of a difference or equivalence between treatment strategies because the included studies were non-comparative and the treatment groups were not necessarily mutually exclusive (Supplementary Figure 7). Overall, reinfection estimates should be interpreted cautiously, reporting variability and differences in follow-up duration. Egger's test did not indicate statistically significant funnel plot asymmetry (intercept −0.58, 95% CI −1.51–0.35; *P* = .2447), and visual inspection did not demonstrate clear asymmetry (Supplementary Figure 8). No random-effects outliers were identified. Given the limited number of studies and clinical heterogeneity across cohorts, the assessment of small-study effects should nevertheless be interpreted cautiously. Comprehensive influence diagnostics, including leave-one-out analysis, standardized residuals, Cook's distance, DFBETAS, leverage, and heterogeneity contribution measures, showed that although some studies were more influential, the leave-one-out pooled estimates remained broadly stable (Supplementary Figure 9).

**Figure 4.**
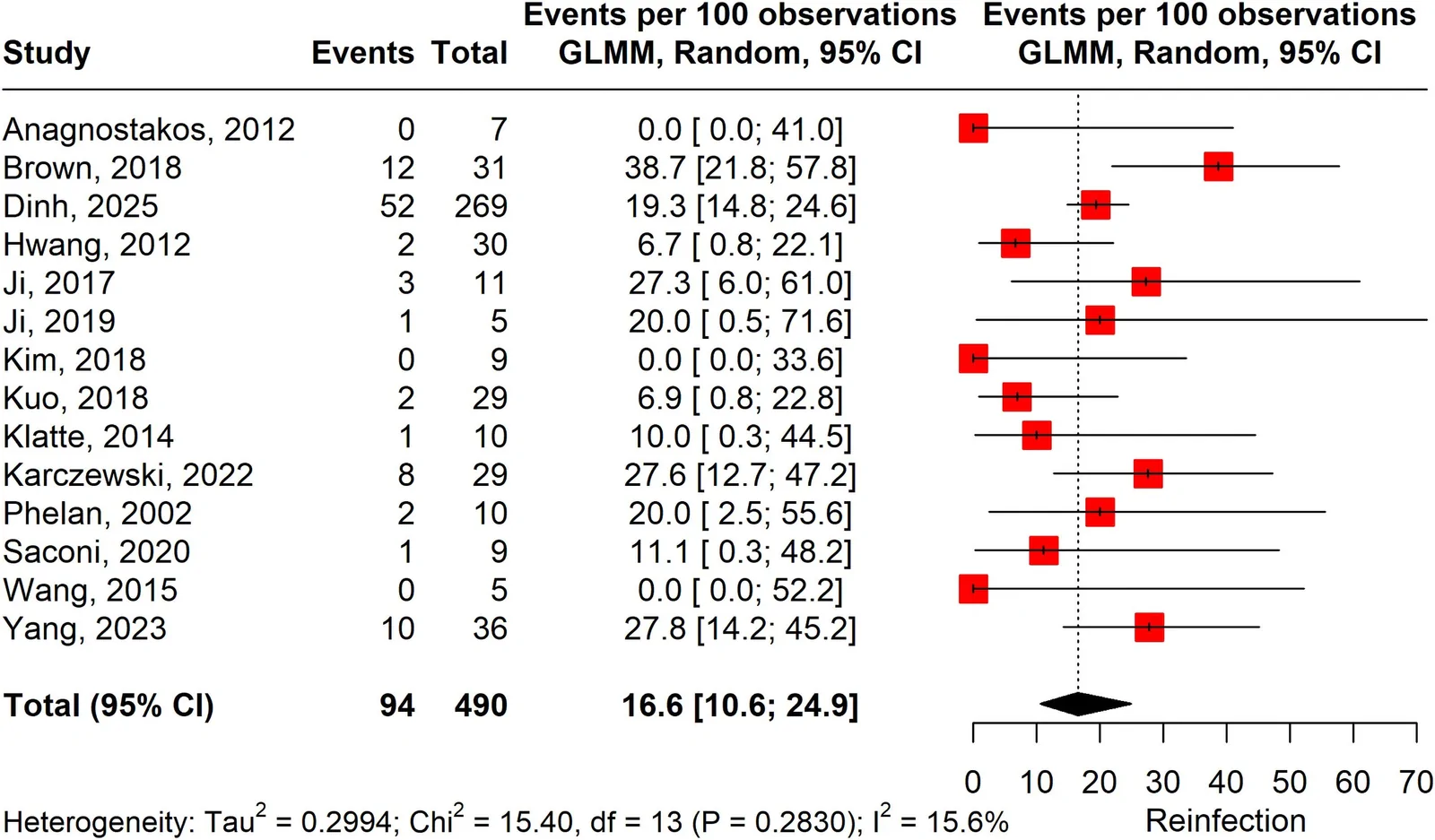
Forest plot of pooled reinfection rates for fungal periprosthetic joint infection (fPJI) using a random-effects generalized linear mixed-effects model (GLMM). The analysis included 14 cohort studies comprising 490 observations and 94 reinfections. The pooled reinfection rate was 16.6% (95% CI, 10.6–24.9), with low heterogeneity (I^2^ = 15.6%).

#### Mortality

The mortality meta-analysis included 9 cohort studies with 157 observations and 15 deaths. The pooled mortality rate was 9.6% (95% CI 5.8–15.2) with minimal heterogeneity (I^2^ = 0%) (Supplementary Figure 10).

Formal assessment of publication bias was not performed because fewer than 10 studies were available. Outlier detection, meta-regression, and subgroup analysis were also not performed for the same reason. Leave-one-out analysis showed that sequential omission of individual studies did not materially change the pooled mortality estimate, with the pooled proportion remaining broadly stable and I^2^ remaining 0% in the leave-one-out analysis; the corresponding leave-one-out influence plot is provided in Supplementary Figure 11.

### Meta-regression Analyses

Meta-regression was performed using publication year and follow-up duration as prespecified moderators to explore between-study heterogeneity. For treatment success, no significant association was found with follow-up duration (k = 25; QM *P* = .3985) or publication year (k = 31; QM *P* = .0552). For treatment failure, neither publication year (k = 27; QM *P* = .1543) nor follow-up duration (k = 22; QM *P* = .4842) was significant. For reinfection, neither publication year (k = 14; QM *P* = .1729) nor follow-up duration (k = 11; QM *P* = .1083) was a significant moderator. Meta-regression was not performed for mortality because too few studies were available (Supplementary Table 5).

### Quality Assessment

Overall quality assessment is summarized in Supplementary Tables 3 and 4. Among the 42 cohort studies, NOS assessments suggested variable methodological quality. Most studies fulfilled several selection and outcome criteria, including microbiological ascertainment of fungal infection and adequate follow-up duration. However, interpretation was limited by the retrospective design of most studies, incomplete follow-up in some cohorts, and inconsistent reporting of outcomes such as reinfection and mortality. Because many studies were non-comparative cohorts, the comparability domain was frequently not applicable and was therefore not considered a major determinant of study quality. Outcome definitions also varied substantially across studies. Therefore, NOS assessments should be interpreted as descriptive measures of methodological quality rather than definitive risk-of-bias classifications. No studies were excluded based on quality assessment, but these methodological limitations support cautious interpretation of the pooled estimates. Case reports and case series showed variable quality and were included only in the qualitative synthesis.

## DISCUSSION

This systematic review and meta-analysis evaluated outcomes of fungal PJI. Cohort meta-analysis showed an overall treatment success rate of about 66.5%, with pooled failure, reinfection, and mortality rates of 36.5%, 16.6%, and 9.6%, respectively. Pooled estimates varied across surgical strategies, with less favorable outcomes reported in studies including DAIR. Reinfection estimates were reported separately for the exchange strategies; however, these findings are descriptive and should not be interpreted as comparative effectiveness. Sensitivity analyses showed that pooled estimates were generally stable. Meta-regression showed no statistically significant associations of publication year or follow-up duration with treatment success, treatment failure, or reinfection. Given the limited number of studies, reporting variability, and clinical heterogeneity, the pooled reinfection estimate should be interpreted cautiously. Overall, fungal PJI remains associated with poor outcomes, and current evidence does not show clear superiority of one exchange strategy over another.

A key clinical question is whether 1-stage exchange can achieve outcomes comparable to 2-stage exchange in fungal PJI. Studies reporting DAIR showed lower success and higher failure rates; however, these findings are highly susceptible to confounding by indication and differences in case selection. Patients selected for DAIR may differ substantially from those undergoing exchange arthroplasty with respect to infection chronicity, comorbidity burden, surgical candidacy, and other clinical factors. Therefore, these descriptive subgroup findings should not be interpreted as evidence of inferior effectiveness of DAIR relative to exchange arthroplasty. Importantly, the pooled estimates observed for 1-stage and 2-stage exchange should not be interpreted as evidence of equivalence, given treatment-selection bias, confounding by indication, and the lack of standardized selection criteria or adjusted comparative analyses. Still, the pooled estimates and low heterogeneity in several 1-stage subgroups suggest that 1-stage approaches may achieve acceptable outcomes in selected cases; however, this observation is based on descriptive data and should not be interpreted as equivalence with 2-stage exchange.

Egger's test demonstrated statistically significant funnel plot asymmetry for the treatment success analysis (*P* = .0281), suggesting the possibility of small-study effects. This finding should be interpreted with caution, as funnel plot asymmetry may arise from several factors, including publication bias, selective reporting, differences in study methodology, and clinical heterogeneity. Given the rarity of fungal PJI and the predominance of small retrospective cohorts, the potential influence of small-study effects on the pooled estimates cannot be excluded.

Sensitivity analyses provide additional reassurance that pooled estimates for success and failure are robust. For reinfection, Egger's test did not indicate significant funnel plot asymmetry, although this finding should be interpreted cautiously given the limited number of studies and heterogeneity across cohorts. Meta-regression did not identify follow-up duration as a statistically significant moderator of reinfection (*P* = .1083). Accordingly, any contribution of follow-up duration to variability in reported reinfection rates remains uncertain. Our reinfection estimates are broadly consistent with prior systematic reviews. Gonzalez et al reported recurrence rates ranging from approximately 18%–30% depending on joint location, whereas Shang et al reported more variable recurrence rates across treatment strategies, with values approaching 30%–50%. These differences highlight the heterogeneity of study populations and treatment approaches in fungal PJI [6, 10].

Mortality estimates were based on fewer studies, showed minimal heterogeneity, and remained stable in leave-one-out analyses. However, because mortality reporting was incomplete across cohorts and definitions of attributable mortality varied, these findings should be interpreted as all-cause mortality during the reported follow-up period.

Meta-regression findings showed study-level rather than patient-level associations. Follow-up duration was not significantly associated with reinfection (*P* = .1083), although differences in follow-up and outcome reporting may still contribute to between-study variability in reported reinfection rates. Future prospective studies should use time-to-event analyses to better define reinfection risk over time.

Outcome definitions also varied substantially across studies, including differences in how treatment success, failure, and reinfection were defined. Also, diagnostic criteria varied across studies and included Musculoskeletal Infection Society (MSIS), European Bone and Joint Infection Society (EBJIS) criteria, culture-based definitions, and author-defined approaches. This can affect which patients are classified as having fungal PJI and may influence reported outcomes. In the surgical literature, treatment success in PJI has often been defined as eradication of infection, absence of further surgery for infection, no infection-related mortality, and no requirement for long-term antimicrobial suppression [55]. However, these criteria were not consistently applied across fungal PJI studies, which complicates comparisons across cohorts and may affect reported treatment success rates. Variation in outcome definitions can substantially affect reported treatment success rates, as previously demonstrated in studies evaluating PJI outcome definitions [56–58]. Using standardized outcome definitions together with structured functional or patient-reported outcomes would allow more comprehensive and comparable assessment of treatment outcomes of fungal PJI in future prospective studies [57, 58].

Overall, these results support several priorities for the field: standardized diagnostic criteria, uniform definitions of success, failure, reinfection, transparent follow-up reporting, and prospective multicenter cohorts, ideally with comparative designs and adjustment for confounding, to better define the optimal surgical strategy and antifungal regimen for fungal PJI.

Exchange arthroplasty is the most reported surgical strategy in the literature, although current evidence does not clearly prove superiority of different stage exchange arthroplasty over another. However, this should not be interpreted as evidence of equivalence, as the available data are derived primarily from retrospective, non-comparative studies and may be influenced by treatment-selection bias and lack of adjusted analyses. Studies reporting DAIR had less favorable pooled outcome estimates; however, this is likely to reflect case selection and should be interpreted cautiously. Management should remain individualized and multidisciplinary.

This study has several limitations. Outcome definitions varied across studies, and fungal PJI is rare, so only a small number of cohort studies were available for meta-analysis, especially for reinfection and mortality. Most studies were retrospective and non-comparative, with surgical decisions based on clinical judgment rather than direct comparison. It should also be noted that many included cohort studies reported fungal PJI cases as subsets within larger heterogeneous patient populations or retrospective case series, rather than as dedicated prospective cohorts, which may limit the internal validity and comparability of reported outcomes. Antifungal regimens were also heterogeneous and inconsistently reported, diagnostic delay was rarely described, and most included studies originated from high-income countries, which may limit the generalizability of these findings to low- and middle-income settings, where access to diagnostic resources, surgical options, and antifungal therapies may differ.

Case reports and case series were included for completeness. Quality assessment findings were used to inform interpretation of the results rather than to exclude studies. Although a predefined protocol was developed and internally approved, it was not prospectively registered, which may limit transparency. Antifungal regimens were highly heterogeneous and inconsistently reported across studies, including substantial variation in agent selection, route of administration, and duration of therapy, which limited meaningful synthesis and interpretation. Additionally, the inclusion of English-language studies only may introduce language bias. Importantly, the included studies were retrospective and non-comparative, and subgroup analyses were based on reported outcome observations that could be patient-, joint-, or procedure-level, with potential overlap between groups. In addition, risk-of-bias assessment was limited by the predominance of single-arm observational cohorts, for which some NOS domains were not applicable. As a result, these findings should be interpreted as descriptive and do not provide evidence of comparative effectiveness or equivalence between treatment strategies.

Despite these limitations, this study has important strengths. To our knowledge, it is the first systematic review to formally meta-analyze cohort-level fungal PJI outcomes and describe pooled outcomes across DAIR, 1-stage, and 2-stage exchange.

Overall, these findings support the need for larger multicenter studies with standardized outcome definitions and better reporting of surgical and antifungal treatment. Until stronger comparative data are available, management should remain individualized. The pooled outcomes reported for 1-stage and 2-stage exchange should be viewed as descriptive rather than evidence of equivalence, while DAIR outcomes should be interpreted cautiously but may still help guide clinical decision-making.

## CONCLUSIONS

This systematic review and meta-analysis reports outcomes for fungal PJI, showing moderate treatment success but substantial risks of failure, reinfection, and mortality. Descriptive subgroup analyses provided separate pooled estimates for 1-stage exchange, 2-stage exchange, and DAIR. These findings should not be interpreted as evidence of comparative effectiveness or equivalence between treatment strategies because of the retrospective, non-comparative evidence base, potential treatment-selection bias, and overlap between treatment groups. Overall, these findings are descriptive and should not be interpreted as evidence of superiority or equivalence between treatment strategies, so management should remain individualized and multidisciplinary. Future prospective, ideally multicenter, comparative studies with standardized diagnostic criteria, outcome definitions, and detailed reporting of antifungal therapy are urgently needed to provide more definitive guidance for optimal management.

## Supplementary Material

ofag657_Supplementary_Data

## Supplementary Data

Supplementary materials are available at *Open Forum Infectious Diseases* online. Consisting of data provided by the authors to benefit the reader, the posted materials are not copyedited and are the sole responsibility of the authors, so questions or comments should be addressed to the corresponding author.
