# Supplementary material for: Fungal Periprosthetic Joint Infection: A Systematic Review and Meta-analysis of Treatment Approaches and Clinical Outcomes
Source: Open Forum Infect Dis. 2026 Oct 8;13(10):ofag657. doi: 10.1093/ofid/ofag657 (PMC13678226; doi:10.1093/ofid/ofag657)
Supplement: Supplementary file 1 [file ofag657_supplementary_data.docx]

**Supplementary materials**

**Table of Contents**

1. **Search Strategies**
   - OVID
   - SCOPUS
2. **Supplementary Tables**
   - Table S1. Characteristics included case reports and case series studies
   - Table S2**.** Detailed outcome per treatment strategies for included cohort studies
   - Table S3. Quality Assessment of Cohort Studies (NOS)
   - Table S4. Quality Assessment of Case Reports and Case Series (Murad Tool)
   - Table S5. Meta-regression Results
3. **Supplementary Figures**
   - Figure S1: Forest Plot: Treatment Success Subgroup analysis
   - Figure S2. Funnel Plot: Treatment Success
   - Figure S3. Influence Diagnostics: Treatment Success
   - Figure S4: Forest Plot: Treatment Failure Subgroup analysis
   - Figure S5. Funnel Plot: Treatment Failure
   - Figure S6. Influence Diagnostics: Treatment Failure
   - Figure S7: Forest Plot: Reinfection Subgroup analysis
   - Figure S8. Funnel Plot: Reinfection
   - Figure S9. Influence Diagnostics: Reinfection
   - Figure S10: Forest Plot: Mortality analysis
   - Figure S11. Influence Diagnostics: Mortality

**Search Strategies**

**OVID**

Database(s): Ovid MEDLINE(R) 1946 to Present and Epub Ahead of Print, In-Process & Other Non-Indexed Citations and Ovid MEDLINE(R) Daily, EBM Reviews - Cochrane Central Register of Controlled Trials October 2025, Embase 1974 to 2025 November 17
Search Strategy:

| **#** | **Searches** |
| --- | --- |
| 1 | ((exp Prosthesis-Related Infections/ or exp prosthesis infection/) and (joint* or hip* or knee* or shoulder* or elbow or elbows or ankle or ankles).ti,ab,hw,kw,kf.) or PJI.ti. |
| 2 | Arthroplasty, Replacement/ or Arthroplasty, Replacement, Ankle/ or Arthroplasty, Replacement, Elbow/ or Arthroplasty, Replacement, Hip/ or Arthroplasty, Replacement, Knee/ or Arthroplasty, Replacement, Shoulder/ or exp Joint Prosthesis/ or (arthroplasty or arthroplasties).ti,ab,hw,kw,kf. or (THA or TKA).ti. or (joint* adj2 (implant* or prosthe*)).ti,ab,hw,kw,kf. |
| 3 | 1 or 2 |
| 4 | exp Mycoses/ or (aspergillus or aspergillosis or candida or candidal or candidemia or candidiasis or cryptococcus or cryptococcosis or dematiaceous or fungal or fungi or fungus or mucor or mucormycosis or mycotic or mycoses or mycosis or non-candida or non-candidal or rhizopus or trichosporon or yeast* or zygomycetes or zygomycosis).ti,ab,hw,kw,kf. |
| 5 | 3 and 4 |
| 6 | ((prosthe* or periprosthe* or peri-prosthe* or peri-implant* or arthroplast* or implant or implants or joint-replacement*) adj10 (aspergillus or aspergillosis or candida or candidal or candidemia or candidiasis or cryptococcus or cryptococcosis or dematiaceous or fungal or fungi or fungus or mucor or mucormycosis or mycotic or mycoses or mycosis or non-candida or non-candidal or rhizopus or trichosporon or yeast* or zygomycetes or zygomycosis)).ti,ab,hw,kw,kf. |
| 7 | 5 or 6 |
| 8 | Treatment Outcome/ or outcome assessment/ |
| 9 | "Follow-Up Studies"/ |
| 10 | exp antifungal agent/dt or exp Antifungal Agents/dt, pc, tu, th or antifungal therapy/ |
| 11 | Prosthesis Retention/ or prosthesis loosening/ |
| 12 | Debridement/ |
| 13 | Reoperation/ or revision arthroplasty/ or two-stage revision/ |
| 14 | Reinfection/ |
| 15 | "Postoperative Complications"/ or "prosthesis complication"/ |
| 16 | "treatment-outcome*".ti,ab. |
| 17 | (((antifungal* or anti-fungal*) adj5 (therap* or treat* or manag* or drug* or medication*)) or (amputat* or aseptic-revision or "combination-therapy" or complication* or DAIR or DOOR or debridement or death or endpoint* or end-point* or eradication or failure or failed or fusion or (implant* adj2 (retention or retain*)) or "infection-control" or (joint* adj2 function*) or mortality or recur* or resect* or relaps* or re-infection or reinfection or reoperat* or re-operat* or revision or spacer-retention or 2-Stage or "two-stage")).ti,ab,hw,kw,kf. or (after or cure or effect* or follow-up or outcome* or rate or rates or risk or success* or incidence or evidence or guideline*).ti. |
| 18 | or/8-17 |
| 19 | 7 and 18 |
| 20 | exp Prosthesis-Related Infections/co, dt, ep, pc, su, th or exp prosthesis infection/co, dt, ep, su, th |
| 21 | joints/ or elbow joint/ or hip joint/ or knee joint/ or shoulder joint/ or (joint* or hip* or knee* or shoulder* or elbow or elbows or ankle or ankles).ti,ab,hw,kw,kf. |
| 22 | exp Mycoses/ or (aspergillus or aspergillosis or candida or candidal or candidemia or candidiasis or cryptococcus or cryptococcosis or dematiaceous or fungal or fungi or fungus or mucor or mucormycosis or mycotic or mycoses or mycosis or non-candida or non-candidal or rhizopus or trichosporon or yeast* or zygomycetes or zygomycosis).ti,ab,hw,kw,kf. |
| 23 | 20 and 21 and 22 |
| 24 | 19 or 23 |
| 25 | limit 24 to (conference abstract or editorial or erratum or note or addresses or autobiography or bibliography or biography or blogs or comment or dictionary or directory or interactive tutorial or interview or lectures or legal cases or legislation or news or newspaper article or overall or patient education handout or periodical index or portraits or published erratum or video-audio media or webcasts or conference review or clinical trial protocol) [Limit not valid in Ovid MEDLINE(R),CCTR,Embase; records were retained] |
| 26 | 24 not 25 |
| 27 | (exp animals/ or exp nonhuman/) not exp humans/ |
| 28 | ((alpaca or alpacas or algae* or amphibian or amphibians or animal or animals or antelope or armadillo or armadillos or avian or baboon or baboons or bats or beagle or beagles or bee or bees or bird or birds or bison or bovine or buffalo or buffaloes or buffalos or bulldog* or "c elegans" or "Caenorhabditis elegans" or camel or camels or canine or canines or canis or carp or cats or catfish or cattle or chamaeleo* or chameleon* or chick or chicken or chickens or chicks or chimp or chimpanze or chimpanzees or chimps or cow or cows or "D melanogaster" or "dairy calf" or "dairy calves" or deer or dog or dogs or donkey or donkeys or drosophila or "Drosophila melanogaster" or duck or duckling or ducklings or ducks or equid or equids or equine or equines or feline or felines or ferret or ferrets or finch or finches or fish or flatworm or flatworms or fox or foxes or frog or frogs or "fruit flies" or "fruit fly" or "G mellonella" or "Galleria mellonella" or geese or gerbil or gerbils or goat or goats or goose or gorilla or gorillas or groundhog or groundhogs or hamster or hamsters or hare or hares or heifer or heifers or horse or horses or iguana or iguanas or insect or insects or jellyfish or kangaroo or kangaroos or kitten or kittens or "laboratory animal*" or lagomorph or lagomorphs or lamb or lambs or lemur or lemurs or lemuridae or llama or llamas or macaque or macaques or macaw or macaws or marmoset or marmosets or mice or minipig or minipigs or mink or minks or monkey or monkeys or mouse or mule or mules or muskrat or muskrats or nematode or nematodes or newt or newts or octopus or octopuses or orangutan or "orang-utan" or orangutans or "orang-utans" or oxen or parrot or parrots or pig or pigeon or pigeons or piglet or piglets or pigs or porcine or primate or primates or poultry or quail or rabbit or rabbits or rat or rats or reptile or reptiles or rodent or rodents or ruminant or ruminants or salmon or sheep or shrimp or slug or slugs or swine or tamarin or tamarins or tilapia or tilapias or toad or toads or trout or urchin or urchins or vole or voles or waxworm or waxworms or weasel or weasels or wolf or wolves or worm or worms or wrass* or xenopus or "zebra fish" or zebrafish) not (human or humans or patient or patients)).ti,ab,hw,kw,kf. |
| 29 | (rat or rats or mice or mouse or murine or pig or pigs or porcine or swine or dog or dogs).ti. |
| 30 | or/27-29 |
| 31 | 26 not 30 |
| 32 | 31 not (metaanalysis or metanalysis or "meta analysis" or "systematic review").ti. |
| 33 | 32 not ("systematic review" or "meta-analysis" or review).pt. |
| 34 | limit 33 to english language |
| 35 | remove duplicates from 34 |

**SCOPUS**

| 1 | ( TITLE-ABS-KEY ( ( prosthe* OR periprosthe* OR peri-prosthe* OR peri-implant* OR arthroplast* OR implant OR implants OR joint-replacement* OR PJI OR TKA OR THA ) W/5 ( aspergillus OR aspergillosis OR candida OR candidal OR candidemia OR candidiasis OR cryptococcus OR cryptococcosis OR dematiaceous OR fungal OR fungi OR fungus OR mucor OR mucormycosis OR mycotic OR mycoses OR mycosis OR non-candida OR non-candidal OR rhizopus OR trichosporon OR yeast* OR zygomycetes OR zygomycosis ) ) ) AND ( TITLE-ABS-KEY ( ( antifungal* OR anti-fungal* ) W/5 ( therap* OR treat* OR manag* OR drug* OR medication* ) ) OR TITLE-ABS-KEY ( amputat* OR aseptic-revision OR "combination-therapy" OR complication* OR DAIR OR DOOR OR debridement OR death OR endpoint* OR end-point* OR eradication OR failure OR failed OR fusion OR ( implant* W/2 ( retention OR retain* ) ) ) OR TITLE-ABS-KEY ( "infection-control" OR ( joint* W/2 function* ) OR mortality OR recur* OR resect* OR relaps* OR re-infection OR reinfection OR reoperat* OR re-operat* OR revision OR spacer-retention OR 2-Stage OR "two-stage" ) OR TITLE ( after OR cure OR effect* OR follow-up OR outcome* OR rate OR rates OR risk OR success* OR incidence OR evidence ) ) |
| --- | --- |
| 2 | ( TITLE-ABS-KEY ( ( alpaca OR alpacas OR amphibian OR amphibians OR animal OR animals OR antelope OR armadillo OR armadillos OR avian OR baboon OR baboons OR beagle OR beagles OR bee OR bees OR bird OR birds OR bison OR bovine OR buffalo OR buffaloes OR buffalos OR "c elegans" OR "Caenorhabditis elegans" OR camel OR camels OR canine OR canines OR carp OR cats OR cattle OR chick OR chicken OR chickens OR chicks OR chimp OR chimpanze OR chimpanzees OR chimps OR cow OR cows OR "D melanogaster" OR "dairy calf" OR "dairy calves" OR deer OR dog OR dogs OR donkey OR donkeys OR drosophila OR "Drosophila melanogaster" OR duck OR duckling OR ducklings OR ducks OR equid OR equids OR equine OR equines OR feline OR felines OR ferret OR ferrets OR finch OR finches OR fish OR flatworm OR flatworms OR fox OR foxes OR frog OR frogs OR "fruit flies" OR "fruit fly" OR "G mellonella" OR "Galleria mellonella" OR geese OR gerbil OR gerbils OR goat OR goats OR goose OR gorilla OR gorillas OR hamster OR hamsters OR hare OR hares OR heifer OR heifers OR horse OR horses OR insect OR insects OR jellyfish OR kangaroo OR kangaroos OR kitten OR kittens OR lagomorph OR lagomorphs OR lamb OR lambs OR llama OR llamas OR macaque OR macaques OR macaw OR macaws OR marmoset OR marmosets OR mice OR minipig OR minipigs OR mink OR minks OR monkey OR monkeys OR mouse OR mule OR mules OR nematode OR nematodes OR octopus OR octopuses OR orangutan OR "orang-utan" OR orangutans OR "orang-utans" OR oxen OR parrot OR parrots OR pig OR pigeon OR pigeons OR piglet OR piglets OR pigs OR porcine OR primate OR primates OR quail OR rabbit OR rabbits OR rat OR rats OR reptile OR reptiles OR rodent OR rodents OR ruminant OR ruminants OR salmon OR sheep OR shrimp OR slug OR slugs OR swine OR tamarin OR tamarins OR toad OR toads OR trout OR urchin OR urchins OR vole OR voles OR waxworm OR waxworms OR worm OR worms OR xenopus OR "zebra fish" OR zebrafish ) AND NOT ( human OR humans OR patient OR patients ) ) ) |
| 3 | 1 AND NOT 2 |
| 4 | 3 AND ( LIMIT-TO ( DOCTYPE , "ar" ) ) AND ( LIMIT-TO ( LANGUAGE , "English" ) ) |
| 5 | INDEX(embase) OR INDEX(medline) OR PMID(0* OR 1* OR 2* OR 3* OR 4* OR 5* OR 6* OR 7* OR 8* OR 9*) |
| 6 | 4 AND NOT 5 **Supplementary Tables** **Table S1**. Characteristics included case reports and case series. C. = *Candida*, DAIR = debridement, antibiotics, and implant retention, EBJIS = European Bone and Joint Infection Society, f/u = follow-up, fx = fracture, N = number of patients, N/A = not applicable, NR = not reported, OA = osteoarthritis, RA = rheumatoid arthritis, USA = United States of America, UK = United Kingdom. **Author** \| **Country** \| **N** \| **Joint** \| **Age** \| **Etiology for index procedure** \| **Time interval since the index procedure (months)** \| **Diagnosis criteria** \| **Organism** \| **Type of Procedure** \| **Min Follow-up** \| **Outcome**; Acikgoz 2002 ^1^ \| Turkey \| 1 \| Knee \| 70 \| OA \| 9 \| Culture based \| *C. glabrata* \| Arthrodesis \| 7-12 months \| Success; Arbeloa-Gutierrez 2016 ^2^ \| Spain \| 1 \| Knee \| 74 \| OA \| 84 \| NR \| *Coccidioides Immitis* \| Arthrodesis \| NR \| Success; Austen 2013 ^3^ \| The Netherlands \| 1 \| Knee \| 77 \| NR \| 71 \| NR \| *Coccidioides Immitis* \| Fluconazole + Resection arthroplasty \| 3-6 months \| Success; Austin 1992 ^4^ \| USA \| 1 \| Knee \| 80 \| OA \| 27 \| NR \| *Aspergillus fumigatus* \| Amphotericin B \| NR \| Success; Badrul 2000 ^5^ \| Malaysia \| 1 \| Knee \| 59 \| OA \| 17 \| NR \| *C. albicans* \| Two stage exchange \| > 2 years \| Reinfection; Baptista 2020 ^6^ \| Portugal \| 1 \| Shoulder \| 68 \| OA \| 3 \| NR \| *Cryptococcus magnus* \| Fluconazole+Amphotericin B + Debridment and spacer \| NR \| Success; Bartash 2017 ^7^ \| USA \| 1 \| Hip \| 54 \| OA \| 1 \| NR \| *Aspergillus terreus* \| Prosthesis explantation with antifungal cement spacer and systemic antifungal therapy \| 6 months \| Success; Baumann 2001 ^8^ \| USA \| 1 \| Knee \| 31 \| Malignancy \| 41 \| NR \| *Aspergillus fumigatus* \| Fluconazole+Amphotericin B + Three stage exchange \| > 2 years \| Success; Berbari 2021 ^9^ \| USA \| 3 \| Two Knees and one shoulder \| 69 & 75 & 77 \| OA : 2 RA: 1 \| 252 & 8 & 2 \| NR \| *Histoplasma Capsulatum* \| Case 1: DAIR + Itraconazole Case 2: Two stage exchange + Itraconazole Case 3 : Three stage exchange + Itraconazole (Shifted to Posoconazole) \| 7-12 months \| Success :3; BiniViotti 2019 ^10^ \| USA \| 1 \| Knee \| 79 \| OA \| 240 \| 2011 \| *C. lusitaniae* \| Fluconazole+Micafungin + Two stage exchange \| 3-6 months \| Success; Bland 2009 ^11^ \| USA \| 1 \| Knee \| 55 \| Trauma \| 2 \| NR \| *C.albicans* \| Debridement + retention + antifungal therapy → failed Prosthesis removal → required for infection control \| NR \| Failure; Bottagisio 2021 ^12^ \| Italy \| 1 \| Hip \| 75 \| OA \| 348 \| NR \| *C.albicans* \| Two stage exchange \| 7-12 months \| Success; Brooks 1998 ^13^ \| USA \| 1 \| Knee \| 64 \| OA \| 15 \| NR \| *C. parapsilosis* \| DAIR \| > 2 years \| Success; Bruce 2001 ^14^ \| UK \| 2 \| Hip \| 59 \| Case 1: NR Case 2 : Hip fx \| 72 & 24 \| NR \| *C.albicans and C. parapsilosis* \| Two stage exchange \| > 2 years \| Success :2; Burgo 2018 ^15^ \| Argentina \| 1 \| Hip \| 73 \| NR \| 228 \| NR \| *Trichosporon inkin* \| Resection arthroplasty+Spacer+Voriconazole + Two stage exchange (Did not complete next step of two stage) \| > 2 years \| Success; Carrega 2017 ^16^ \| Italy \| 1 \| Hip \| 68 \| NR \| 18 \| NR \| *C.albicans* \| Two stage exchange + anidulafungin \| 13-24 months \| Success; Chiu Wang Kei 2013 ^17^ \| Hong Kong \| 1 \| Hip \| 71 \| Avascular necrosis \| 180 \| NR \| *C. parapsilosis* \| Fluconazole \| > 2 years \| Success; Chong 2022 ^18^ \| Hong Kong \| 1 \| Knee \| 76 \| OA \| 6 \| NR \| *C. parapsilosis* \| Two stage exchange \| > 2 years \| Success; Cifci 2023 ^19^ \| Turkey \| 1 \| Knee \| 72 \| OA \| 20 \| NR \| *C.albicans* \| DAIR + caspofungin + posaconazole \| 3-6 months \| Success; Ciochetto 2021^20^ \| USA \| 1 \| Knee \| 75 \| OA \| 144 \| NR \| *Coccidioides* \| Two-stage exchange + amphotericin B + oral fluconazole (lifelong suppression) \| 6 \| Success; Cobo 2017^21^ \| Spain \| 1 \| Hip \| 77 \| OA \| 96 \| NR \| *C. glabrata* \| Two stage exchange+ caspofungin (8days) and fluconazole 6 months \| 5 \| Success; Cs 2025^22^ \| India \| 1 \| Knee \| 65 \| NR \| 48 \| NR \| *C. parapsilosis* \| Two stage exchange + Fluconazole \| 7-12 months \| Success; Cushing 1997^23^ \| USA \| 1 \| Knee \| 73 \| NR \| 30 \| NR \| *C. parapsilosis* \| Fluconazole \| 7-12 months \| Success; Darouiche 1989^24^ \| USA \| 4 \| 3 Hip and one Knee \| 63.5 \| NR \| 4 \| NR \| *3 C.albicans one C.tropicalis* \| Resection arthroplasty \| 7-12 months \| Success :4; Deelstra 2013^25^ \| Netherlands \| 1 \| Hip \| 74 \| Trauma \| 12 \| NR \| *C.albicans* \| Three stage exchange \| > 2 years \| Success; DeHart 1995^26^ \| USA \| 1 \| Knee \| 56 \| OA \| 24 \| NR \| *Sporothrix schenckii* \| DAIR + amphotericin B and itraconazole \| > 2 years \| Failure; Dumaine 2008^27^ \| France \| 1 \| Knee \| 72 \| NR \| NR \| NR \| *C. glabrata* \| Arthrodesis \| 13-24 months \| Success; Dunkley 1997^28^ \| UK \| 1 \| Metacarpophalangeal \| 76 \| NR \| 2 \| NR \| *C.albicans* \| Salvage Procedure \| 3-6 months \| Success; Dutronc 2010^29^ \| France \| 7 \| 3 Hip and 4 Knee \| 72 \| NR \| NR \| NR \| *4 C.albicans 1 C. guilliermondii and 2 C. parapsilosis* \| One with DAIR and 3 with Two stage exchnage and one with Arthrodesis surgery and one patient with resection arthroplasty, one with suppressive therapy \| > 2 years \| Two stage (1 Success, 2 failure) DAIR (success); antifungal suppressive therapy (persistant pain); Arthrodesis (bacterial reinfection/failure), resection arthroplasty (Success).; Evans 1990^30^ \| USA \| 2 \| Hip \| 54 \| Case 1: OA Case 2: RA \| 35 \| NR \| *2 C.albicans* \| Two stage exchange \| > 2 years \| Success :2; Fabry 2005^31^ \| Belgium \| 1 \| Knee \| 74 \| OA \| 72 \| NR \| *C. glabrata* \| DAIR + Voriconazole \| 8 \| Success; Fowler 1998^32^ \| USA \| 1 \| Hip \| 84 \| Trauma \| 156 \| NR \| *Histoplasma capsulatum* \| DAIR + Itraconazole (lifelong) \| > 2 years \| Success; Frank 2025^33^ \| Switzerland \| 2 \| One Hip one Knee \| 56.5 \| Case 1: Avascular necrosis Case 2 : Trauma \| 120 \| NR \| *C.albicans and C. parapsilosis* \| Two stage exchange \| 4 years and 1.5 years \| Success :2; Frieler 2020^34^ \| Germany \| 1 \| Knee \| 58 \| NR \| 12 \| MSIS 2018 \| *C. parapsilosis* \| Three stage exchange \| > 2 years \| Success; Fukasawa 1997^35^ \| Japan \| 1 \| Knee \| 80 \| OA \| 2 \| NR \| *C. parapsilosis* \| DAIR+ Fluconazole \| > 2 years \| Success; Gabriel 2024^36^ \| USA \| 1 \| Shoulder \| 83 \| Rotator cuff arthropathy \| 14 \| NR \| *Cryptococcus neoformans* \| One Stage Exchange \| 13-24 months \| Success; Gautam 2025^37^ \| India \| 1 \| Knee \| 53 \| OA \| 1 \| NR \| *C. parapsilosis* \| DAIR \| 3-6 months \| Success; George 2024^38^ \| India \| 5 \| Hip :2 Knee :3 \| 67.6 \| NR \| 22.8 \| NR \| *5 C. parapsilosis* \| 4 One Stage Revision One DAIR \| > 2 years \| Success :5; George 2021^39^ \| India \| 2 \| Knee \| 64.5 \| NR \| 30 \| NR \| *C. parapsilosis and one* *Candida not specified* \| One Stage Exchange \| > 2 years \| Success :2; Giordani 2023^40^ \| USA \| 2 \| One Hip one Knee \| 34.5 \| OA \| 36 \| NR \| *Coccidioides* \| Two stage exchange and one stage exchange \| 13-24 months \| Success :2; Giovanoulis 2022^41^ \| France \| 1 \| Knee \| 74 \| OA \| 120 \| NR \| *C. lusitaniae* \| Arthrodesis \| 7-12 months \| Success; Goodman 1983^42^ \| USA \| 2 \| Hip :1 Knee :1 \| 64 \| OA \| 16 \| NR \| *C. tropicalis and torulopsis glabrata* \| Arthrodesis \| NR \| Success = 1. Failure =1; Gottesman-Yekutieli 2011^43^ \| Israel \| 1 \| Hip \| 56 \| RA \| 30 \| NR \| *Pseudallescheria boydii* \| Two stage exchange \| > 2 years \| Success; Graw 2010^44^ \| USA \| 2 \| Knee \| 68.5 \| OA \| 120 & 3 \| NR \| *2 C.albicans* \| Two stage exchange \| > 2 years \| Success :2; Hall 2012^45^ \| UK \| 1 \| Hip \| 60 \| Trauma \| NR \| NR \| *C. glabrata* \| Girdlestone (resection arthroplasty) \| NR \| Success; Halstead 2025^46^ \| UK \| 1 \| Knee \| 68 \| NR \| 108 \| NR \| *Coccidioides* \| Fluconazole and Amphotericin B and Voriconazole \| NR \| Success; Hekman 2025^47^ \| USA \| 1 \| Hip \| 75 \| OA \| 324 \| NR \| *C. glabrata* \| DAIR \| 7-12 months \| Failure; Henriquez 2025^48^ \| USA \| 1 \| Shoulder \| NR \| NR \| NR \| NR \| *C.albicans* \| Resection arthroplasty \| NR \| Success; Hirsch 1996^49^ \| USA \| 1 \| Hip \| 53 \| OA \| 408 \| NR \| *Aureobasidium* \| Two stage exchange \| 13-24 months \| Success; Hnatow 2025^50^ \| USA \| 1 \| Knee \| 71 \| NR \| NR \| NR \| *C.albicans* \| Two stage exchange \| > 2 years \| Success; Ishak 2023^51^ \| Malaysia \| 1 \| Knee \| 85 \| NR \| 84 \| NR \| *Cyberlindnera fabianii* \| Resection arthroplasty + fluconazole \| NR \| Success; Jenny 2016^52^ \| France \| 2 \| Hip :1 Knee :1 \| 65.5 \| NR \| 42 \| NR \| *2 C.albicans* \| One Stage Exchange \| > 2 years \| Success: 2; Johannsson 2009^53^ \| USA \| 1 \| Hip \| 84 \| NR \| NR \| NR \| *Cryptococcus neoformans* \| Fluconazole+Amphotericin B \| 10 \| Failure; Keck 2025^54^ \| USA \| 1 \| Hip \| NR \| NR \| NR \| NR \| *C. Parapsilosis* \| Rezafungin \| NR \| Success; Keuning 2019^55^ \| the Netherlands \| 1 \| Knee \| 72 \| OA \| 108 \| NR \| *C. parapsilosis* \| Two stage exchange \| 13-24 \| Success; Kobayashi 2021^56^ \| USA \| 1 \| Finger arthroplasty \| 74 \| OA \| 1 \| NR \| *Aspergillus terreus* \| One stage exchange + Voriconazole \| > 2 years \| Success; Koutserimpas 2022^57^ \| Greece \| 1 \| Knee \| 72 \| OA \| 120 \| NR \| *C. parapsilosis* \| Two stage exchange \| > 2 years \| Success; Koutserimpas 2018^58^ \| Greece \| 1 \| Knee \| 68 \| OA \| 180 \| NR \| *C. glabrata* \| Voriconazole \| 3-6 \| Success; Kuner 2019^59^ \| Switzerland \| 1 \| Knee \| 87 \| OA \| 22 \| NR \| *C. guilliermondii* \| Two Stage Exchange \| 13-24 \| Success; Kurmis 2021^60^ \| Australia \| 1 \| Knee \| 70 \| OA \| 66 \| MSIS 2018 \| *Aspergillus fumigatus* \| Two stage exchange \| 12 \| Success; Kuthan 2023^61^ \| Poland \| 1 \| Knee \| 74 \| OA \| 36 \| EBJIS 2021 \| *Aspergillus clavatus* \| Resection arthroplasty with antibiotic cement spacer and Itraconazole \| NR \| Success; Lackner 2011^62^ \| Austria \| 1 \| Knee \| 61 \| OA \| 1 \| NR \| *Pseudallescheria Apiosperma* \| Arthrodesis \| > 2 years \| Success; Lazzarini 2004^63^ \| Italy \| 1 \| Hip \| 63 \| OA \| 7 \| NR \| *C.albicans* \| Resection arthroplasty + Amphotericin B \| > 2 years \| Success; Lejko-Zupanc 2005^64^ \| Slovenia \| 1 \| Hip \| 73 \| Trauma \| 72 \| NR \| *C. glabrata* \| Two stage exchange \| > 2 years \| Success; Lerch 2003^65^ \| Germany \| 1 \| Knee \| 78 \| OA \| 36 \| NR \| *C.albicans* \| Arthrodesis and resection arthroplasty \| > 2 years \| Success; Leylabadlo 2016^66^ \| Iran \| 1 \| Knee \| 59 \| OA \| 0.5 \| NR \| *Malassezia* \| Fluconazole+Voriconazole+Amphotericin B \| NR \| Success; Li 2023^67^ \| China \| 1 \| Knee \| 56 \| OA \| 0.25 \| NR \| *C. parapsilosis* \| DAIR \| > 2 years \| Success; Lidder 2013^68^ \| UK \| 1 \| Hip \| 76 \| OA \| 60 \| NR \| *C. tropicalis* \| Two stage exchange \| 13-24 months \| Success; Lim 1986^69^ \| USA \| 1 \| Shoulder \| 70 \| Trauma \| 3 \| NR \| *C.albicans* \| Resection arthroplasty + Amphotericin B \| > 2 years \| Success; Lin 2022^70^ \| China \| 1 \| Hip \| 76 \| Trauma \| 9 \| NR \| *C.albicans* \| DAIR and two stage exchange \| 7-12 months \| Success; Liu 2024^71^ \| China \| 1 \| Knee \| 62 \| OA \| 4 \| NR \| *C. parapsilosis* \| DAIR+ Voriconazole \| 13-24 months \| Success; Longfellow 2025^72^ \| USA \| 1 \| Knee \| 60 \| RA \| 3.5 \| NR \| *Fusarium* \| DAIR and Arthrodesis \| 13-24 months \| Success; MacLean 2015^73^ \| USA \| 1 \| Knee \| 56 \| NR \| NR \| NR \| *Blastomyces dermatitidis* \| Two stage exchange \| > 2 years \| Success; Mafrachi 2021^74^ \| Jordan \| 1 \| Knee \| 60 \| OA \| 8 \| NR \| *C. parapsilosis* \| Two stage exchange \| 7-12 months \| Success; Marcomini 2023^75^ \| Ireland \| 1 \| Hip \| 63 \| OA \| 72 \| NR \| *C. parapsilosis* \| DAIR \| NR \| Failure; Meiyappan 2019^76^ \| USA \| 1 \| Knee \| 57 \| NR \| 96 \| NR \| *Histoplasma capsulatum* \| Three stage exchange \| > 2 years \| Success; Mello Lopez 2023^77^ \| Brazil \| 1 \| Knee \| 77 \| Other \| NR \| NR \| *Trichosporon species 1* \| Surgical debridement, subsequent amputation \| 2 month \| Failure; Merrer 2001^78^ \| France \| 1 \| Hip \| 81 \| OA \| 144 \| NR \| *C.albicans* \| Fluconazole \| 11 \| Success; Morimoto 2021^79^ \| Japan \| 1 \| Hip \| 73 \| OA \| 240 \| NR \| *C.albicans* \| DAIR and two stage exchange \| > 2 years \| Success; Murata 2020^80^ \| Japan \| 1 \| Hip \| 60 \| OA \| 1 \| NR \| *C. tropicalis* \| One Stage Exchange + Fluconazole \| > 2 years \| Success; Nadarevic 2025^81^ \| USA \| 1 \| Shoulder \| 60 \| Trauma \| 14 \| NR \| *C. glabrata* \| Itraconazole +Micafungin + Surgizal removal \| > 2 years \| Reinfection and then death; Nowbakht 2017^82^ \| USA \| 1 \| Knee \| 77 \| OA \| 156 \| NR \| *Histoplasma capsulatum* \| Two stage exchange \| > 2 years \| Success; Oenning 2020^83^ \| Germany \| 1 \| Knee \| 55 \| OA \| 1 \| NR \| *C.albicans* \| Three stage exchange \| > 2 years \| Success; Ornell 2019^84^ \| USA/Canada \| 1 \| Elbow \| 57 \| Trauma \| 6 \| NR \| *C. parapsilosis* \| DAIR and resection arthroplasty \| > 2 years \| Death; Pasticci 2019^85^ \| Italy \| 1 \| Hip \| 71 \| OA \| 72 \| NR \| *C. glabrata* \| Two stage exchange \| > 2 years \| Success; Patel 2025^86^ \| USA \| 1 \| Knee \| 60 \| OA \| 85 \| NR \| *C. auris* \| One Stage Exchange \| 3-6 months \| Success; Pozzi 2023^87^ \| Switzerland \| 2 \| Hip :1 Knee :1 \| 73 & 57 \| NR \| 36 & NR \| NR \| *C. glabrata and C. tropicalis* \| One with DAIR and another one with two stage exchange and resection arthroplasty \| 13-24 months \| Success : 2; Presman 2014^88^ \| Denmark \| 2 \| Hip \| 63 and 59 \| NR \| N/A \| NR \| *C.albicans* \| One Stage Exchange and Two Stage exchange \| 3-6 months \| Success & failure; Radha 2012^89^ \| UK \| 1 \| Shoulder \| 71 \| RA \| 36 \| NR \| *C.albicans* \| Excision arthroplasty (resection arthroplasty) with systemic antifungal therapy \| NR \| Success; Rajme-Lopez 2023^90^ \| Mexico \| 1 \| Knee \| 67 \| OA \| 108 \| NR \| *Histoplasma capsulatum* \| Resection arthroplasty \| > 2 years \| Success; Ramamohan 2001^91^ \| UK \| 1 \| Hip \| 65 \| OA \| 60 \| NR \| *C. glabrata* \| Two stage exchange and amphotericin B+ 5 flucytosine \| > 2 years \| Success; Reddy 2013^92^ \| India \| 1 \| Knee \| 62 \| OA \| 24 \| NR \| *C. tropicalis* \| Two stage exchange \| > 2 years \| Success; Sarzaeem 2022^93^ \| Iran \| 1 \| Knee \| 75 \| Trauma \| 8 \| NR \| *C. tropicalis* \| Two stage exchange \| 12 \| Success; Sebastian , 2018 ^94^ \| India \| 1 \| Hip \| 53 \| Trauma \| 48 \| NR \| *C. tropicalis* \| Two stage exchange \| > 2 years \| Success; Selmon 1998^95^ \| United Kingdom \| 1 \| Knee \| 75 \| OA \| 84 \| NR \| *C. glabrata* \| One Stage Exchange \| > 2 years \| Success; Shah 2015^96^ \| USA \| 1 \| Hip \| 61 \| OA \| 6 \| NR \| *Cryptococcus neoformans* \| Amphotericin B+ Fluconazole \| 13-24 months \| Success; Simonian 1997^97^ \| USA \| 1 \| Knee \| 76 \| OA \| 42 \| NR \| *C. albicans* \| DAIR + Ketoconazole \| > 2 years \| Success; Skedros 2014^98^ \| USA \| 1 \| Shoulder \| 58 \| Trauma \| 0.75 \| NR \| *C. glabrata* \| Resection arthroplasty 🡪 failure 🡪 chronic suppressive antifungal therapy (fluconazole)🡪 failure 🡪 salvage surgery + suppressive therapy 🡪 success. \| > 2 years \| Success; Song 2020^99^ \| South Korea \| 1 \| Knee \| 75 \| NR \| 32 \| NR \| *C. pelliculosa* \| Two stage exchange \| > 2 years \| Success; Springer 2012^100^ \| USA \| 1 \| Shoulder \| 65 \| RA \| 84 \| NR \| *C. albicans* \| DAIR \| 7-12 months \| Success; Tabliago 2022^101^ \| USA \| 1 \| Knee \| 60 \| OA \| 96 \| NR \| *Acremonium strictum* \| Two stage exchange \| 7-12 months \| Success; Sharma 2024^102^ \| India \| 1 \| Knee \| 59 \| OA \| 84 \| 2011 \| *C. tropicalis* \| Two stage exchange \| 12 \| Success; Tiwari 2024^103^ \| India \| 1 \| Hip \| 33 \| Trauma \| 48 \| NR \| *C. albicans* \| Two stage exchange \| 7-12 months \| Success; Tummala 2023^104^ \| USA \| 1 \| Hip \| 64 \| NR \| 192 \| NR \| *Bipolaris* \| DAIR \| > 2 years \| Success; Tunkel 1993^105^ \| USA \| 1 \| Knee \| 37 \| OA \| 72 \| NR \| *C. parapsilosis* \| Two stage exchange 🡪 failure 🡪 suppressive therapy with fluconazole 🡪 failure 🡪 amputation \| 13-24 months \| Success; Unfried 2022^106^ \| Germany \| 1 \| Hip \| 73 \| OA \| 36 \| NR \| *C. albicans* \| Two stage exchange and resection arthroplasty \| > 2 years \| Success; Vergison 2020^107^ \| Belgium \| 1 \| Hip \| 73 \| OA \| 1 \| EBJIS 2018 \| *C. parapsilosis* \| Two stage exchange and resection arthroplasty \| 3-6 months \| Success; Wada 1998^108^ \| Japan \| 1 \| Knee \| 77 \| OA \| 0.5 \| NR \| *C. parapsilosis* \| DAIR \| > 2 years \| Success; White 1995^109^ \| USA \| 1 \| Knee \| 64 \| OA \| 9 \| NR \| *C. parapsilosis* \| Resection arthroplasty+ Amphotericin B + Itraconazole \| 13-24 months \| Failure; Williams 2022^110^ \| USA \| 1 \| Knee \| 54 \| NR \| 18 \| MSIS 2018 \| *Histoplasma capsulatum* \| Two stage exchange+ Amphotericin B \| 13-24 months \| Success; Wiwattanawarang 2014^111^ \| Thailand \| 2 \| Knee \| 71.5 \| OA \| 72 & 48 \| NR \| *C. albicans: 2* \| Two stage exchange and resection arthroplasty \| 9 & NR \| Success = 2; Wu 2014^112^ \| Taiwan \| 1 \| Hip \| 47 \| NR \| 120 \| NR \| *Acremonium strictum* \| Two stage exchange \| 7-12 months \| Success; Wu 2011^113^ \| Taiwan \| 1 \| Knee \| 72 \| OA \| 144 \| NR \| *C. albicans* \| Three stage exchange \| 7-12 months \| Success; Xiang 2018^114^ \| China \| 1 \| Knee \| 54 \| OA \| 0.25 \| NR \| *C. albicans* \| Fluconazole + Voriconazole \| 3-6 months \| Success; Xin 2020^115^ \| China \| 1 \| Knee \| 62 \| OA \| 20 \| NR \| *C. parapsilosis* \| Two stage exchange and Arthrodesis and resection arthroplasty \| > 2 years \| Success; Yang 2001^116^ \| Taiwan \| 1 \| Knee \| 68 \| OA \| 16 \| NR \| *C. parapsilosis* \| Two stage exchange + fluconazole \| > 2 years \| Success; Younkin 1984^117^ \| USA \| 1 \| Hip \| 75 \| OA \| 0 \| NR \| *C. parapsilosis* \| Resection arthroplasty \| > 2 years \| Success; Zuo 2015^118^ \| China \| 1 \| Knee \| 73 \| OA \| 1 \| NR \| *Trichosporon asahii* \| DAIR \| > 2 years \| Success **Table S2.** Detailed outcome per treatment strategies for included cohort studies. ^a^ This study provided patient level data and overall success and failure calculated based on patient level data. ^b^ Authors did not report successful treatment but they mentioned infection eradicated in all cases. ^c^ Gao et al. reported sequential treatment strategies. Five joints initially underwent DAIR; five failed DAIR subsequently underwent two-stage revision. Outcomes were analyzed according to each treatment episode, resulting in treatment-specific counts that exceed the number of unique patients/joints. **Abbreviations:** DAIR = debridement, antibiotics, and implant retention, DIAGNOSIS = diagnostic definition/criteria used by study authors, f/u = follow-up, fPJI = fungal periprosthetic joint infection, N = number of patients, NR = not reported, PJI = periprosthetic joint infection, THA = total hip arthroplasty, TKA = total knee arthroplasty, USA = United States of America **Author year** \| **Inclusion criteria** \| **N** \| **Joint** \| **Treatment** \| **Outcome**; Anagnostakos 2021^119^ \| Patients who underwent revision surgery due to septic reason \| 3 \| Hip:1 Knee:2 \| DAIR:1 Two-stage exchange:2 \| DAIR: 1 death; Two-stage exchange: 2 patients (1 lost to follow-up, 1 treatment failure).; Anagnostakos, 2012^120^ \| Patients with fungal PJI who underwent two-stage exchange \| 7 \| Hip:4 Knee:3 \| Two-stage exchange:7 \| Two-stage exchange: 7 successes; Azzam 2009^121^ \| Patients who were diagnosed with a fungal PJI \| 31 \| Hip:14 Knee:17 \| DAIR:7 (all failed and 5 converted to two stage 2 medical therapy) Arthrodesis:3 Two stage exchange :19, Resection + spacer without successful second stage: 10 patients + all the patients get 6 weeks antifungal therapy (Fluconazole + Caspofungin and Amphotericin B) \| DAIR: 7 (0 success); Two-stage exchange: 19 (9 success, 10 failure (one of them died)); Definitive resection: 10 (no reimplantation); Baecker 2021^122^ \| Patients with fungal PJI underwent three-stage revision \| 18 \| Hip:11 Knee:7 \| Three-stage exchange:18 \| Three-stage exchange: 18 (16 success, 2 failure (one of them died); Brown 2018^123^ \| Patients >18 years old with fungal PJI \| 31 \| Hip:13 Knee:18 \| Two-stage, DAIR, one-stage exchange retention, resection arthroplasty, antifungal suppression, spacer retained in situ \| 16/31 Success, 15/31 failure ^a^ (including 12/31 reinfections), Two-stage: 7 success, 7 failure. one stage exchange: 1 success, DAIR: 4 failures, retained spacers: 1 success and 1 failure, Treatment-specific outcome was not reported for the remaining 10 patients; Burr 2022^124^ \| Patients >18 years with PJI \| 1 \| Hip:1 \| Fluconazole + Amoxicillin \| Successful treatment; Dinh 2025^125^ \| Patients with confirmed PJI \| 269 \| Hip:146 Knee:118 Shoulder:4 Ankle:1 \| DAIR:96 One-stage exchange:76 Two-stage exchange:78 Resection arthroplasty:7 Salvage:4 Amputation:2 (strategy for 6 patients not reported) \| Overall: Success:156/269, failure 113/269, 52/269 Reinfection DAIR: 96 (45 success, 51 failure); One-stage exchange: 76 (51 success, 25 failure); 2-stage exchange: 78 (54 success, 24 failure); Definitive resection: 7 (3 success, 4 failure); salvage: 4 (2 success, 2 failure) and amputations (1 success and 1 failure), 6/269 treatment-based outcome NR,; Eckers 2021^126^ \| NR \| 1 \| Knee:1 \| DAIR and then Amputation \| DAIR: Failure; Enz 2021^127^ \| Patients underwent revision due to PJI \| 13 \| NR \| Two-stage: 7; DAIR: 1; Resection/drain: 5 \| Two-stage: 7 (6 success, 1 failure); DAIR: 1 (1success); Resection: resection/drain: 3 success (2 were treated with caspofungin and 1 no fungicide), 2 failure (one was treated with fluconazole and the other no antifungals); Escola-Verge 2018^128^ \| Patients with Candida spp. PJI \| 43 \| Hip:26 Knee:16 Shoulder:1 \| DAIR: 15; Resection 20 (The remaining 8 patients were not evaluated (6 death unrelated to FPJI, 1 did not finish treatment, 1 amputation due to bacterial superinfection) \| Overall : 17/35 Success, 18/35 Failure DAIR: 15 (4 success, 11 failure); Prosthesis removal: 20 (13 success, 7 failure); Gao 2018^129^ \| Patients with fungal PJI \| 17 \| Hip:5 Knee:13 (17 patients but 18 joints) \| Two-stage revision: 18 (all joints); Initial DAIR: 5 (all failed and converted to two-stage) \| 13/23 Success, 10/23 failure Two-stage revision: 18 (13 success, 5 failure) DAIR (5 failures and converted to two stage) ^c^; Geng 2016^130^ \| Patients with fungal PJI \| 8 \| Hip:4 Knee:4 \| Two-stage exchange: 7; Resection arthroplasty: 1 \| Two-stage exchange: 7 (6 success, 1 failure); Resection arthroplasty: 1 (success); Gonzalez 2024^131^ \| Patients with fungal PJI and 2 years f/u \| 10 \| Knee:10 \| DAIR: 4; DAIR-plus: 4; Two-stage revision: 1; Chronic suppression only (no surgery): 1 \| Overall: Success:1/10, Failure 9/10, DAIR: 4 (0 success); DAIR-plus: 4 (1 success, 3 failure); Two-stage: 1 (0 success → amputation), chronic suppression: death; Hartman 2022^132^ \| Patients with two stage exchange and 2 years f/u \| 2 \| NR \| Two-stage exchange:2 \| Two-stage exchange: 2 (2 success, 0 failure); Herndon 2023^133^ \| Patients known to have a fungal PJI \| 41 \| Hip:22 Knee:19 \| Two-stage exchange (resection + spacer): 16; Repeat spacer exchange (infected spacer / repeat 2-stage): 10; DAIR (modular exchange): 4; Irrigation & debridement alone: 2; Arthrodesis: 2; Amputation: 2; One-stage exchange: 1; Rapid two-stage exchange: 1; reimplantation: 3 \| Overall outcome reported: 41 (20 success, 21 failure); Hwang 2012^134^ \| Patients with fungal PJI \| 28 \| Knee:30 (Two bilateral) \| Two-stage exchange:30 \| Two-stage revision: 30 (28 success, 2 reinfection); Jeon 2023^135^ \| NR \| 1 \| Knee:1 \| DAIR:1 \| Successful treatment; Ji 2022^136^ \| Patients with PJI and treated with One-stage exchange \| 7 \| NR \| One Stage exchange :7 \| One-stage: 7 (4 success, 3 failure); Ji 2019^137^ \| Patients who had undergone one-stage revision for chronically infected THA \| 5 \| Hip:5 \| One-stage exchange: 5 \| Success: 4 and 1 relapse; Ji 2017^138^ \| Patients with chronic fungal PJI \| 11 \| Hip:4 Knee:7 \| One-stage exchange:11 \| 7 success, 3 relapse/reinfection, 1 death; Kanar, 2023^139^ \| Patients with infected TKA \| 1 \| Knee:1 \| Arthrodesis 1 \| Success; Karczewski 2022^140^ \| Patients with Candida PJI \| 29 \| Hip: 14; Knee: 15 \| Predominantly two- or multi-stage exchange; minority DAIR, one-stage, or permanent resection (counts NR) \| Overall: 8/29 reinfection, 21/29 not extractable; Kazimoglu 2015^141^ \| NR \| 1 \| Hip:1 \| DAIR:1 \| Successful treatment; Kim 2018^142^ \| Patients with fungal PJI who underwent two-stage exchange \| 9 \| Knee:9 \| Two-stage exchange:9 \| Two-stage exchange: 9 (9 success, 0 failure); Kim 2021^143^ \| Patients underwent second two-stage exchange \| 6 \| Knee:6 \| Two-stage exchange:6 \| Two-stage exchange: 6 (2 success, 4 failure); Klatte 2014^144^ \| Patients with confirmed fungal PJI \| 10 \| Hip:6 Knee:4 \| One-stage exchange:10 \| One-stage exchange: 10 (9 success, 1 failure); Kuo 2018^145^ \| PJI patients with ≥2 positive fungal cultures \| 29 \| Hip:14 Knee:15 \| DAIR: 7, One-stage exchange: 3, Two-stage exchange arthroplasty: 19 \| DAIR: 7 (2 success, 5 failure); One-stage revision: 3 (1 success, 2 failure); Two-stage exchange: 19 (11 success, 8 failure (two of them died).; Lachman 2018^146^ \| Patients with acute hematogenous ankle PJI \| 1 \| Ankle:1 \| DAIR:1 \| Failure; Lee 2024^147^ \| Chronic knee PJI treated with two-stage exchange \| 12 \| Knee:12 \| Two-stage exchange:12 \| 5 success, 7 failure; McCulloch 2023^148^ \| Patients with ≥2 positive deep fungal cultures \| 69 \| Hip:24 Knee:45 \| Mixed procedures including DAIR & One and two stage exchange and suppression and excision and amputations \| Mixed procedures: 33 success, 36 failures (Outcomes are not reported separately for every treatment); Miller 2015^149^ \| Patients with culture- or histology-proven Candida osteoarticular infection \| 8 \| Hip:5 Knee:2 Shoulder:1 \| Surgical debridement with removal of prosthesis, antifungal therapy, reimplantation in selected cases \| 8 success, 0 failure; Phelan 2002^150^ \| Patients with THA& TKA infection due to Candida species \| 10 \| Hip:6 Knee:4 \| Two-stage exchange:10 \| Two-stage exchange: 8 success, 2 reinfection; Saconi 2020^151^ \| Culture-positive Candida PJI \| 11 \| Hip:6 Knee:5 \| DAIR:1 One-stage exchange:4 Two-stage exchange:1 Resection arthroplasty:5 \| Overall : 8/9 Success, 1/9 Failure, 2/11 NR, 1/9 Reinfection, 2/11 NR DAIR: 1 success, 0 failure One-stage exchange: 2 success, 1 failure, 1 NR Two-stage exchange: 1 success, 0 failure Resection arthroplasty: 4 success, 1 NR; Sidhu 2019^152^ \| Hip/knee PJIs with both bacterial and fungal cultures \| 22 \| Hip:8 Knee:14 \| Two Stage exchange: 22 \| Two-stage exchange: 9 success, 13 failure; Starnes 2024^153^ \| Patients with fungal PJI \| 18 \| Hip:9 Knee:9 \| DAIR:4 , One-stage exchange:1 ,Two-stage exchange:7 Girdlestone procedure:1 Stage-one destination:1 Amputation:1 (3/18 treatment strategy NR) \| Outcome was available for 11/18 patients; 7/18 had no reported outcome. Among evaluable patients, 4/11 were successful and 7/11 failed. Two-stage exchange: 3/7 success, 4/7 failure; Theil 2019^154^ \| Culture-proven fungal THA/TKA PJI \| 26 \| Hip:18 Knee:8 \| Two-stage exchange: 23, One-stage exchange: 2, implant retention + antifungal therapy :1 \| Overall: 10/26 Success, 16/26 failure Two-stage exchange: 10 success, 13 failure, One stage exchange: 0 success, 2 failure Implant retention + antifungal therapy: 1 failure; Ueng 2013^155^ \| Hip/knee candidal PJI with >=2 positive cultures \| 16 \| Hip:7 Knee:9 \| Two-stage exchange:16 (12 completed, 4 only first stage completed) \| Two-stage exchange: 8 success, 8 failure (4 of them died); Wang 2020^156^ \| Revision hip/knee with seronegative infection \| 1 \| Knee:1 \| One-stage exchange:1 \| Success; Wang 2015^157^ \| Culture-proven fungal knee PJI \| 5 \| Knee:5 \| Two-stage exchange:5 \| Two-stage exchange: 5 success; Whiteside 2017^158^ \| Infected THA treated with single-stage revision \| 1 \| Hip:1 \| One-stage exchange:1 \| Successful treatment; Xu 2024^159^ \| Patients with fungal PJI \| 8 \| Hip:3 Knee:5 \| Two-stage exchange:6 Knee arthrodesis:2 \| 6/6 Success, 2 Knee arthrodesis: 2 success (Infection eradicated in all cases) ^b^; Yang 2023^160^ \| Fungal TKA PJI treated with two-stage exchange \| 41 \| Knee:41 \| Two-stage exchange & DAIR \| Overall, 26/46 success, 20/46 failure (including 10/46 reinfection), Two-stage exchange: 26/36 success, 10/36 failure (reinfection), DAIR: 10 failures **Table S3.** Quality assessment of cohort studies using the Newcastle–Ottawa Scale (NOS). NA, not applicable. Because many included studies were retrospective, non-comparative single-arm cohorts, comparator-dependent NOS items (selection of the non-exposed cohort and comparability of cohorts) were assessed only when a relevant comparison group was available; otherwise, these domains were designated NA. An asterisk (*) indicates that the corresponding NOS criterion was met, whereas a dash (–) indicates that an applicable criterion was not met. Assessments are presented at the domain level without calculation of an overall NOS score or classification into low-, moderate-, or high-risk categories. NOS findings should therefore be interpreted as descriptive indicators of methodological quality rather than definitive measures of risk of bias. \| Selection \| \| \| \| Comparability \| Outcome \| \|; **Study** \| *1) Representativeness of the Exposed Cohort \| *2) Selection of the Non-Exposed Cohort \| *3) Ascertainment of Exposure \| *4) Demonstration That Outcome of Interest Was Not Present at Start of Study \| **1) Comparability of Cohorts on the Basis of the Design or Analysis \| *1) Assessment of Outcome \| *2)Was Follow-Up Long Enough for Outcomes to Occur \| *3) Adequacy of Follow Up of Cohorts; **Anagnostakos 2021** \| * \| NA \| * \| * \| NA \| * \| * \| -; **Anagnostakos 2012** \| * \| NA \| * \| * \| NA \| * \| * \| *; **Azzam 2009** \| * \| NA \| * \| * \| NA \| * \| * \| *; **Baecker 2021** \| * \| NA \| * \| * \| NA \| * \| * \| *; **Brown 2018** \| * \| NA \| * \| * \| NA \| * \| * \| *; **Burr 2022** \| * \| NA \| * \| * \| NA \| * \| * \| *; **Dinh 2025** \| * \| NA \| * \| * \| NA \| * \| * \| *; **Eckers 2021** \| * \| * \| * \| * \| * \| * \| * \| *; **Enz 2021** \| * \| * \| * \| * \| * \| * \| - \| -; **Escola-Verge 2018** \| * \| NA \| * \| * \| NA \| * \| * \| *; **Gao 2018** \| * \| NA \| * \| * \| NA \| * \| * \| *; **Geng 2016** \| * \| NA \| * \| * \| NA \| * \| * \| *; **Gonzalez 2024** \| * \| NA \| * \| * \| NA \| * \| * \| *; **Hartman 2022** \| * \| NA \| * \| * \| NA \| * \| * \| *; **Herndon 2023** \| * \| NA \| * \| * \| NA \| * \| * \| -; **Hwang 2012** \| * \| NA \| * \| * \| NA \| * \| * \| *; **Jeon 2023** \| * \| NA \| * \| * \| NA \| * \| * \| *; **Ji 2022** \| * \| NA \| * \| * \| NA \| * \| * \| *; **Ji 2017** \| * \| NA \| * \| * \| NA \| * \| * \| *; **Ji 2019** \| * \| NA \| * \| * \| NA \| * \| * \| -; **Kanar 2023** \| * \| NA \| * \| * \| NA \| * \| * \| *; **Karczewski 2022** \| * \| NA \| * \| * \| NA \| * \| * \| *; **Kazimoglu 2015** \| * \| NA \| * \| * \| NA \| * \| * \| *; **Kim 2018** \| * \| NA \| * \| * \| NA \| * \| * \| *; **Kim 2021** \| * \| NA \| * \| * \| NA \| * \| * \| *; **Klatte 2014** \| * \| NA \| * \| * \| NA \| * \| * \| -; **Kuo 2018** \| * \| NA \| * \| * \| NA \| * \| * \| *; **Lachman 2018** \| * \| NA \| * \| * \| NA \| * \| * \| *; **Lee 2024** \| * \| NA \| * \| * \| NA \| * \| * \| *; **McCulloch 2023** \| * \| NA \| * \| * \| NA \| * \| * \| -; **Miller 2015** \| * \| NA \| * \| * \| NA \| * \| * \| -; **Phelan** \| - \| NA \| * \| * \| NA \| * \| * \| *; **Saconi** \| * \| NA \| * \| * \| NA \| * \| * \| -; **Sidhu 2019** \| * \| NA \| * \| * \| NA \| * \| * \| *; **Starnes** \| * \| NA \| * \| * \| NA \| * \| - \| -; **Theil 2019** \| * \| NA \| * \| * \| NA \| * \| * \| *; **Ueng 2013** \| * \| NA \| * \| * \| NA \| * \| * \| *; **Wang 2020** \| * \| NA \| * \| * \| NA \| * \| * \| *; **Wang 2015** \| * \| NA \| * \| * \| NA \| * \| * \| *; **Whiteside 2017** \| - \| NA \| * \| * \| NA \| * \| * \| *; **Xu 2024** \| * \| NA \| * \| * \| NA \| * \| * \| *; **Yang 2023** \| * \| NA \| * \| * \| NA \| * \| * \| * |

**Table S4. Quality Assessment of case reports and case series studies using Murad tool**

| Study | Selection Q1 Does the patient(s) represent the whole experience of the investigator (centre) or is the selection method Unclear to the extent that other patients with similar presentation may not have been reported? | Selection Q2 Was the exposure adequately ascertained? | Ascertainment Q3 Was the outcome adequately ascertained? | Ascertainment Q4 Was follow-up long enough for outcomes to occur? (minimum 6 month follow up for FPJI) | Causality Q5 Is there sufficient detail to determine that other alternative causes that may explain the observation were ruled out? | Causality Q6 Was there a challenge/rechallenge phenomenon? | Causality Q7 Was there a dose-response effect? | Reporting Q8 Is the case(s) described with sufficient details to allow other investigators to replicate the research or to allow practitioners to make inferences related to their own practice? |
| --- | --- | --- | --- | --- | --- | --- | --- | --- |
| Acikgoz 2002 | Unclear | Yes | Yes | Yes | Unclear | NA | NA | Yes |
| Arbeloa-Gutierrez 2016 | Unclear | Yes | Yes | Yes | Yes | NA | NA | Yes |
| Austen 2013 | Unclear | Yes | Yes | Yes | Yes | NA | NA | Yes |
| Austin 1992 | Unclear | Yes | Yes | No | Yes | NA | NA | Yes |
| Badrul 2000 | Unclear | Yes | Yes | Yes | Yes | NA | NA | Yes |
| Baptista 2020 | Unclear | Yes | Yes | Yes | Yes | NA | NA | Yes |
| Bartash 2017 | Unclear | Yes | Yes | no | Yes | NA | NA | Yes |
| Baumann 2001 | Unclear | Yes | Yes | Yes | Yes | NA | NA | Yes |
| Berbari 2021 | Unclear | Yes | Yes | Yes | Yes | NA | NA | Yes |
| BiniViotti 2019 | No | Yes | Yes | Yes | Yes | NA | NA | Yes |
| Bland 2009 | Unclear | Yes | Yes | No | Yes | Yes | NA | Yes |
| Bottagisio 2021 | Unclear | Yes | Yes | Yes | Yes | NA | NA | Yes |
| Brooks 1998 | Unclear | Yes | Yes | Yes | Yes | NA | NA | Yes |
| Bruce 2001 | Unclear | Yes | Yes | Yes | Yes | NA | NA | Yes |
| Burgo 2018 | No | Yes | Yes | Yes | Yes | NA | NA | Yes |
| Carrega 2017 | Unclear | Yes | Yes | Yes | Yes | NA | NA | Yes |
| Chiu 2013 | Unclear | Yes | Yes | Yes | Yes | NA | NA | Yes |
| Chong 2022 | Unclear | Yes | Yes | Yes | Yes | NA | Unclear | Yes |
| Çifci 2023 | Unclear | Yes | Yes | No | Yes | NA | NA | Yes |
| Ciochetto 2021 | Unclear | Yes | Yes | Yes | Yes | Yes | NA | Yes |
| Cobo 2017 | Unclear | Yes | Yes | No | Yes | NA | NA | Yes |
| Cs 2025 | Unclear | Yes | Yes | Yes | Yes | NA | NA | Yes |
| Cushing 1997 | Unclear | Yes | Yes | Yes | Yes | NA | NA | Yes |
| Darouiche 1989 | Unclear | Unclear | Yes | Yes | Unclear | NA | NA | Yes |
| Deelstra 2013 | Unclear | Yes | Yes | Yes | Yes | NA | NA | Yes |
| DeHart 1995 | Unclear | Yes | Yes | Yes | Yes | Yes | NA | Yes |
| Dumaine 2008 | No | Yes | Yes | Yes | Yes | NA | NA | Yes |
| Dunkley 1997 | No | Yes | Yes | Yes | Yes | NA | NA | Yes |
| Dutronc 2010 | Yes | Yes | Yes | Yes | Unclear | NA | NA | Yes |
| Evans 1990 | Unclear | Yes | Yes | Yes | Yes | NA | NA | Yes |
| Fabry 2005 | Unclear | Yes | Yes | No | Yes | Yes | NA | Yes |
| Fowler 1998 | No | Yes | Yes | Yes | Unclear | NA | NA | YES |
| Frank 2025 | No | Yes | Yes | Yes | Unclear | No | No | Yes |
| Frieler 2020 | No | Yes | Yes | Yes | Yes | NA | NA | Yes |
| Fukasawa 1997 | Unclear | Yes | Yes | Yes | Yes | NA | NA | Yes |
| Gabriel 2024 | Unclear | Yes | Yes | Yes | Yes | NA | NA | Yes |
| Gautam 2025 | No | Yes | Yes | No | Yes | NA | NA | Yes |
| George 2024 | Yes | Yes | Yes | Yes | Yes | NA | NA | Yes |
| George 2021 | Unclear | Yes | Yes | Yes | Yes | NA | NA | Yes |
| Giordani 2023 | Unclear | Yes | Yes | Yes | Yes | NA | NA | Yes |
| Giovanoulis 2022 | Unclear | Yes | Yes | Yes | Yes | NA | NA | Yes |
| Goodman 1983 | Unclear | Yes | Yes | No | Yes | NA | NA | Yes |
| Gottesman-Yekutieli 2011 | No | Yes | Yes | Yes | Yes | NA | NA | Yes |
| Graw 2010 | Unclear | Yes | Yes | Yes | Yes | NA | NA | Yes |
| Hall 2012 | No | Yes | Unclear | Unclear | Unclear | NA | NA | Yes |
| Halstead 2025 | No | Yes | Unclear | Unclear | Yes | NA | NA | Yes |
| Hekman 2025 | Unclear | Yes | Unclear | Yes | Yes | NA | NA | Yes |
| Henriquez 2025 | Yes | Yes | Yes | Yes | Yes | NA | NA | Yes |
| Hirsch 1996 | Unclear | Yes | Yes | Yes | Yes | NA | NA | Yes |
| Hnatow 2025 | No | Yes | Yes | Yes | Yes | NA | NA | Yes |
| Ishak 2023 | No | Yes | No | No | Yes | NA | NA | Yes |
| Jenny 2016 | Unclear | Yes | Yes | Yes | Yes | NA | NA | Yes |
| Johannsson 2009 | No | Yes | Yes | Yes | Yes | NA | NA | Yes |
| Keck 2025 | Unclear | Yes | Yes | Unclear | Unclear | NA | NA | No |
| Keuning 2019 | No | Yes | Yes | Yes | Yes | NA | NA | YES |
| Kobayashi 2021 | No | Yes | Yes | Yes | Yes | NA | NA | Yes |
| Koutserimpas 2022 | Unclear | Yes | Yes | Yes | Yes | NA | NA | Yes |
| Koutserimpas 2018 | No | Yes | No | No | Yes | NA | NA | Yes |
| Kuner 2019 | No | Yes | Yes | Yes | No | NA | NA | YES |
| Kurmis 2021 | No | Yes | Yes | Yes | Yes | NA | NA | Yes |
| Kuthan 2023 | Unclear | Yes | Yes | No | No | NA | NA | Yes |
| Lackner 2011 | Unclear | Yes | Yes | Yes | Yes | NA | NA | Yes |
| Lazzarini 2004 | Unclear | Yes | Yes | Yes | Yes | NA | NA | Yes |
| Lejko-Zupanc 2005 | Unclear | Yes | Yes | Yes | Yes | NA | NA | Yes |
| Lerch 2003 | Unclear | Yes | Yes | Yes | Yes | No | Yes | Yes |
| Leylabadlo 2016 | No | Unclear | Unclear | Unclear | Unclear | NA | NA | Unclear |
| Lidder 2013 | No | Yes | Yes | Yes | Unclear | NA | NA | Yes |
| Lim 1986 | Unclear | Yes | Yes | Unclear | Yes | NA | NA | Yes |
| Li 2023 | Unclear | Yes | Yes | Yes | Yes | NA | NA | Yes |
| Lin 2022 | Unclear | Yes | Yes | Yes | Unclear | NA | NA | Yes |
| Liu 2024 | Unclear | Yes | Yes | Yes | Unclear | NA | NA | Yes |
| Longfellow 2025 | No | Yes | Yes | Yes | Yes | NA | NA | Yes |
| MacLean 2015 | No | Yes | Yes | Yes | Yes | NA | NA | Yes |
| Mafrachi 2021 | No | Yes | Yes | Yes | Unclear | NA | NA | Yes |
| Marcomini 2023 | No | Yes | Yes | Yes | Yes | NA | NA | Yes |
| Meiyappan 2019 | No | Yes | Yes | Yes | Yes | NA | NA | Yes |
| MelloLopez 2023 | Yes | Yes | Yes | Yes | Unclear | NA | NA | Yes |
| Merrer 2001 | No | Yes | Yes | Yes | Unclear | NA | NA | Yes |
| Morimoto 2021 | Unclear | Yes | Yes | Yes | Unclear | NA | NA | Yes |
| Murata 2020 | No | Yes | Yes | Yes | Unclear | NA | NA | Yes |
| Nadarevic 2025 | No | Yes | Yes | No | Unclear | NA | NA | Yes |
| Nowbakht 2017 | No | Yes | Yes | Yes | Yes | NA | NA | Yes |
| Oenning 2020 | Unclear | Yes | Yes | Yes | Unclear | NA | NA | Yes |
| Ornell 2019 | No | Yes | Yes | Yes | Unclear | NA | NA | Yes |
| Pasticci 2019 | No | Yes | Yes | Yes | Yes | NA | NA | Yes |
| Patel 2025 | Unclear | Yes | Yes | no | Yes | NA | NA | Yes |
| Pozzi 2023 | Unclear | Yes | Yes | Yes | Yes | NA | NA | Yes |
| Presman 2014 | Unclear | Yes | Yes | no | Unclear | NA | NA | Yes |
| Radha 2012 | Unclear | Yes | Unclear | Unclear | Yes | NA | NA | Yes |
| Rajme-Lopez 2023 | Unclear | Yes | Yes | Unclear | Yes | NA | NA | Yes |
| Ramamohan 2001 | No | Yes | Yes | Yes | Yes | NA | NA | Yes |
| Reddy 2013 | No | Yes | Yes | Yes | Yes | NA | NA | Yes |
| Sarzaeem 2022 | No | Yes | Yes | Yes | Yes | NA | NA | Yes |
| Sebastian 2018 | Unclear | Yes | Yes | Yes | Yes | NA | NA | Yes |
| Selmon 1998 | Unclear | Yes | Yes | Yes | Yes | NA | NA | Yes |
| Shah 2015 | Unclear | Yes | Yes | Yes | Yes | NA | NA | Yes |
| Simonian 1997 | Unclear | Yes | Yes | Yes | Yes | NA | NA | Yes |
| Skedros 2014 | No | Yes | Yes | Yes | Unclear | NA | NA | Yes |
| Song 2020 | No | Yes | Yes | Unclear | Yes | NA | NA | YES |
| Springer 2012 | No | Yes | Yes | Yes | Yes | NA | NA | Yes |
| Tabliago 2022 | No | Yes | Yes | Yes | Yes | NA | NA | Yes |
| Sharma 2024 | No | Yes | Yes | Yes | Unclear | NA | NA | Yes |
| Tiwari 2024 | No | Yes | Yes | Yes | Unclear | NA | NA | Yes |
| Tummala 2023 | No | Yes | Yes | Yes | Yes | NA | NA | Yes |
| Tunkel 1993 | No | Yes | Yes | No | Unclear | NA | NA | Yes |
| Unfried 2022 | No | Yes | Yes | Yes | No | No | No | Yes |
| Vergison 2020 | No | Yes | Yes | Yes | Yes | NA | NA | Yes |
| Wada 1998 | Unclear | Yes | Yes | Yes | Yes | NA | NA | Yes |
| White 1995 | Unclear | Yes | Yes | Unclear | Unclear | NA | NA | Yes |
| Williams 2022 | No | Yes | Yes | Yes | Yes | NA | NA | Yes |
| Wiwattanawarang 2014 | Yes | Yes | Unclear | Unclear | Unclear | NA | NA | Yes |
| Wu 2014 | No | Yes | Yes | Yes | Unclear | NA | NA | Yes |
| Wu 2011 | Unclear | Yes | Yes | Yes | Unclear | NA | NA | Yes |
| Xiang 2018 | Unclear | Yes | Yes | No | Unclear | No | No | Yes |
| Xin 2020 | No | Yes | Yes | Yes | Yes | NA | NA | Yes |
| Yang 2001 | No | Yes | Yes | Yes | Yes | NA | NA | Yes |
| Younkin 1984 | Unclear | Yes | Yes | Yes | Yes | NA | NA | Yes |
| Zuo 2015 | No | Yes | Yes | Yes | Yes | NA | NA | Yes |

**Table S5.** Meta-regression results evaluating publication year and follow-up duration (months) as moderators of pooled outcome estimates (success, failure, reinfection), reporting residual heterogeneity (τ², I², H²) and the moderator test (QM p-value).

| **Outcome** | **Moderator** | **k** | **Residual τ²** | **Residual I² (%)** | **Residual H²** | **P value for moderator (QM)** |
| --- | --- | --- | --- | --- | --- | --- |
| **Treatment success** | Follow-up duration (months) | 25 | 1.3194 | 87.53 | 8.02 | 0.3985 |
| **Treatment success** | Publication year | 31 | 1.0076 | 81.89 | 5.52 | 0.0552 |
| **Treatment failure** | Follow-up duration (months) | 22 | 1.2885 | 88.19 | 8.47 | 0.4842 |
| **Treatment failure** | Publication year | 27 | 1.0935 | 84.06 | 6.27 | 0.1543 |
| **Reinfection** | Follow-up duration (months) | 11 | 0.2029 | 42.30 | 1.73 | 0.1083 |
| **Reinfection** | Publication year | 14 | 0.2040 | 38.67 | 1.63 | 0.1729 |

**Supplementary Figures**

**Supplementary Figure S1.** Descriptive estimates of treatment success stratified by reported treatment strategy among outcome observations from studies of fungal periprosthetic joint infection (fPJI). Because the source studies used mixed units of analysis, the observations may represent patients, joints, or procedures, depending on the source study. The forest plot presents study-level and pooled treatment success proportions with 95% confidence intervals for two-stage exchange, one-stage exchange, and debridement, antibiotics, and implant retention (DAIR). Pooled estimates were calculated using a random-effects generalized linear mixed-effects model (GLMM). The estimates are presented descriptively by reported treatment strategy; no formal statistical test of differences across treatment strategies was performed.

**Supplementary Figure S1 alt text:** Forest plot showing descriptive treatment success estimates stratified by reported treatment strategy among outcome observations from studies of fungal periprosthetic joint infection. Observations may represent patients, joints, or procedures, depending on the source study. The figure includes two-stage exchange, one-stage exchange, and DAIR. Individual study estimates are represented by red squares with horizontal 95% confidence intervals, and pooled estimates are represented by diamonds. Random-effects GLMM pooled treatment success estimates are 69.7% for two-stage exchange, 68.8% for one-stage exchange, and 13.3% for DAIR. No formal statistical test of differences across treatment strategies is displayed.

**Supplementary Figure S2.** Funnel plot for the meta-analysis of overall treatment success in fungal periprosthetic joint infection (fPJI), showing logit-transformed proportions plotted against their standard errors. Egger’s regression test indicated statistically significant funnel plot asymmetry (p = 0.0281)

**Supplementary Figure S2 alt text:** Funnel plot of overall treatment success in fungal PJI, displaying logit-transformed treatment success proportions against their standard errors. Egger’s regression test indicated statistically significant funnel plot asymmetry (p = 0.0281).

**Supplementary Figure S3**. Comprehensive influence diagnostics for the meta-analysis of overall treatment success in fungal periprosthetic joint infection (fPJI). The figure summarizes study-level influence measures, including leave-one-out pooled estimates, standardized residuals, DFBETAS, Cook’s distance, leverage (hat values), and contributions to heterogeneity (Q and I²). While several studies showed relatively greater influence or contribution to heterogeneity, the leave-one-out pooled estimates remained broadly stable across omitting individual studies, indicating that the overall findings were generally robust.

**Supplementary Figure S3 alt text:** Comprehensive influence diagnostics for the meta-analysis of overall treatment success in fungal PJI, displaying study-level influence measures, leave-one-out pooled estimates, standardized residuals, DFBETAS, Cook’s distance, leverage, and contributions to heterogeneity. Although several studies showed greater influence or heterogeneity contributions, leave-one-out pooled estimates remained broadly stable, indicating that the overall treatment success estimate was not materially driven by any single study.

**Supplementary Figure S4.** Descriptive estimates of treatment failure stratified by reported treatment strategy among outcome observations from studies of fungal periprosthetic joint infection (fPJI). Because the source studies used mixed units of analysis, the observations may represent patients, joints, or procedures, depending on the source study. The forest plot presents study-level and pooled treatment failure proportions with 95% confidence intervals for one-stage exchange, two-stage exchange, and debridement, antibiotics, and implant retention (DAIR). Pooled estimates were calculated using a random-effects generalized linear mixed-effects model (GLMM). The pooled treatment failure estimates were 31.2% (95% CI, 22.6–41.3) for one-stage exchange, 34.6% (95% CI, 24.7–46.1) for two-stage exchange, and 86.7% (95% CI, 54.3–97.3) for DAIR. The estimates are presented descriptively by reported treatment strategy; no formal statistical test of differences across treatment strategies was performed.

**Supplementary Figure S4 alt text:** Forest plot showing descriptive treatment failure estimates stratified by reported treatment strategy among outcome observations from studies of fungal periprosthetic joint infection. Observations may represent patients, joints, or procedures, depending on the source study. The figure includes one-stage exchange, two-stage exchange, and DAIR. Individual study estimates are represented by red squares with horizontal 95% confidence intervals, and pooled estimates are represented by diamonds. Random-effects GLMM pooled treatment failure estimates are 31.2% (95% CI, 22.6–41.3) for one-stage exchange, 34.6% (95% CI, 24.7–46.1) for two-stage exchange, and 86.7% (95% CI, 54.3–97.3) for DAIR. No formal statistical test of differences across treatment strategies is displayed.

**Supplementary Figure S5**. Funnel plot for the meta-analysis of overall treatment failure in fungal periprosthetic joint infection (fPJI), showing logit-transformed proportions plotted against their standard errors. Visual inspection did not demonstrate clear funnel plot asymmetry, and Egger’s regression test was not statistically significant (p = 0.1108).

**Supplementary Figure S5 alt text:** Funnel plot of overall treatment failure in fungal PJI, displaying logit-transformed treatment failure proportions against their standard errors. The study estimates show no clear funnel plot asymmetry, and Egger’s regression test was not statistically significant (p = 0.1108).

**Supplementary Figure S6**. Comprehensive influence diagnostics for the meta-analysis of overall treatment failure in fungal periprosthetic joint infection (fPJI). The figure summarizes study-level influence measures, including leave-one-out pooled estimates, standardized residuals, DFBETAS, Cook’s distance, leverage (hat values), and contributions to heterogeneity (Q and I²). Although some studies showed relatively greater influence, the leave-one-out pooled estimates remained broadly stable across omission of individual studies, indicating that the overall treatment failure findings were generally robust.

**Supplementary Figure S6 alt text:** Comprehensive influence diagnostics for the meta-analysis of overall treatment failure in fungal PJI, displaying study-level influence measures, leave-one-out pooled estimates, standardized residuals, DFBETAS, Cook’s distance, leverage, and contributions to heterogeneity. Although some studies showed greater influence or heterogeneity contributions, leave-one-out pooled estimates remained broadly stable, indicating that the overall treatment failure estimate was not materially driven by any single study.

**Supplementary Figure S7.** Descriptive estimates of reinfection stratified by reported treatment strategy among outcome observations from studies of fungal periprosthetic joint infection (fPJI). Because the source studies used mixed units of analysis, the observations may represent patients, joints, or procedures, depending on the source study. The forest plot presents study-level and pooled reinfection proportions with 95% confidence intervals for two-stage exchange and one-stage exchange. Pooled estimates were calculated using a random-effects generalized linear mixed-effects model (GLMM). The pooled reinfection estimate was 12.6% (95% CI, 5.2–27.4) for two-stage exchange and 19.2% (95% CI, 8.2–38.7) for one-stage exchange. The estimates are presented descriptively by reported treatment strategy; no formal statistical test of differences across treatment strategies was performed.

**Supplementary Figure S7 alt text:** Forest plot showing descriptive reinfection estimates stratified by reported treatment strategy among outcome observations from studies of fungal periprosthetic joint infection. Observations may represent patients, joints, or procedures, depending on the source study. The figure includes two-stage exchange and one-stage exchange. Individual study estimates are represented by red squares with horizontal 95% confidence intervals, and pooled estimates are represented by diamonds. Random-effects GLMM pooled reinfection estimates are 12.6% (95% CI, 5.2–27.4) for two-stage exchange and 19.2% (95% CI, 8.2–38.7) for one-stage exchange. No formal statistical test of differences across treatment strategies is displayed.

**Supplementary Figure S8.** Funnel plot assessing small-study effects for the meta-analysis of reinfection following surgical treatment of fungal periprosthetic joint infection (fPJI). The plot displays study-level logit-transformed reinfection proportions plotted against their standard errors. Visual inspection did not demonstrate clear funnel plot asymmetry, and Egger’s regression test was not statistically significant (intercept −0.58, 95% CI −1.51 to 0.35; p = 0.2447).

**Supplementary Figure S8 alt text:** Funnel plot of reinfection following surgical treatment of fungal PJI, displaying study-level logit-transformed reinfection proportions against their standard errors. The study estimates show no clear funnel plot asymmetry, and Egger’s regression test was not statistically significant (p = 0.2447).

**Supplementary Figure S9.** Comprehensive influence diagnostics for the meta-analysis of reinfection following surgical treatment of fungal periprosthetic joint infection (fPJI). The figure summarizes study-level influence measures, including leave-one-out pooled estimates, standardized residuals, DFBETAS, Cook’s distance, leverage (hat values), and contributions to heterogeneity (Q and I²). The diagnostics were used to assess whether the pooled reinfection estimate was disproportionately influenced by any individual study.

**Supplementary Figure S9 alt text:** Comprehensive influence diagnostics for the meta-analysis of reinfection in fungal PJI, displaying study-level influence measures, leave-one-out pooled estimates, standardized residuals, DFBETAS, Cook’s distance, leverage, and contributions to heterogeneity. The figure evaluates the effect of sequential omission of individual studies on the pooled reinfection estimate and heterogeneity.

**Supplementary Figure S10.** Forest plot showing the pooled treatment mortality rate for fungal PJI across included cohort studies.

**Supplementary Figure S10 alt text:** Forest plot showing mortality rates across included cohort studies of fungal PJI. The random-effects GLMM pooled mortality estimate was 9.6% (95% CI, 5.8–15.2), with no observed heterogeneity (I² = 0%).

**Supplementary Figure S11**. Leave-one-out influence diagnostics for the meta-analysis of mortality in fungal periprosthetic joint infection (fPJI). The panels display study-level diagnostic measures, including sequential omission analyses, standardized residuals, DFBETAS, Cook’s distance, leverage (hat values), contributions to heterogeneity (Q), and changes in the pooled mortality proportion and I² after omission of individual studies. Across sequential omission of each study, the pooled mortality estimate remained broadly stable, with I² remaining 0%, suggesting no marked single-study effect on the overall mortality estimate**.**

**Supplementary Figure S11 alt text:** Comprehensive influence diagnostics for the meta-analysis of mortality in fungal PJI, displaying study-level influence measures, leave-one-out pooled estimates, standardized residuals, DFBETAS, Cook’s distance, leverage, and contributions to heterogeneity. Leave-one-out pooled mortality estimates remained broadly stable, with I² remaining 0%, indicating that the overall mortality estimate was not materially driven by any single study.

**References:**

1.	Açikgöz ZC, Şayli U, Avci S, Doğruel H, Gamberzade Ş. An Extremely Uncommon Infection: Candida glabrata Arthritis after Total Knee Arthroplasty. *Scandinavian Journal of Infectious Diseases.* 2002/01/01 2002;34(5):394–396.

2.	Arbeloa-Gutierrez L, Kuberski T, Johnson SM, Sagastibelza I, Alaez JI, Pappagianis D. Reactivation of coccidioidomycosis: a prosthetic joint infection in Spain. *European Journal of Clinical Microbiology & Infectious Diseases.* 2016/02/01 2016;35(2):183–186.

3.	Austen S, van der Weegen W, Verduin CM, van der Valk M, Hoekstra HJ. Coccidioidomycosis Infection of a Total Knee Arthroplasty in a Nonendemic Region. *The Journal of Arthroplasty.* 2013/02/01/ 2013;28(2):375.e313–375.e315.

4.	Austin KS, Testa NN, Luntz RK, Greene JB, Smiles S. Aspergillus infection of total knee arthroplasty presenting as a popliteal cyst: Case report and review of the literature. *The Journal of Arthroplasty.* 1992/09/01/ 1992;7(3):311–314.

5.	Badrul B, Ruslan G. Candida albicans infection of a prosthetic knee replacement: a case report. *Med J Malaysia.* Sep 2000;55 Suppl C:93–96.

6.	Baptista M, Sevivas N, Ferreira NV, Fardilha L, Varanda P, Mateus C. Cryptococcus magnus Periprosthetic Shoulder Infection: A Case Report. *JBJS Case Connector.* 2020;10(4).

7.	Bartash R, Guo Y, Pope JB, et al. Periprosthetic hip joint infection with Aspergillus terreus: A clinical case and a review of the literature. *Medical Mycology Case Reports.* 2017/12/01/ 2017;18:24–27.

8.	Baumann PA, Cunningham B, Patel NS, Finn HA. Aspergillus fumigatus infection in a mega prosthetic total knee arthroplasty: Salvage by staged reimplantation with 5-year follow-up. *The Journal of Arthroplasty.* 2001/06/01/ 2001;16(4):498–503.

9.	Berbari HE, Gurram P, Mahmood M, Deziel PJ, Walker RC, Razonable RR. Prosthetic Joint Infections Due to Histoplasma capsulatum: A Report of 3 Cases. *Mayo Clinic Proceedings: Innovations, Quality & Outcomes.* 2021/02/01/ 2021;5(1):225–229.

10.	Bini Viotti J, Corzo-Pedroza M, Gonzales Zamora JA. Prosthetic joint infection caused by Candida lusitaniae: report of a unique case. *Acta Clinica Belgica.* 2019/07/04 2019;74(4):286–291.

11.	Bland CM, Thomas S. Micafungin plus Fluconazole in an Infected Knee with Retained Hardware due to Candida albicans. *Annals of Pharmacotherapy.* 2009/03/01 2009;43(3):528–531.

12.	Bottagisio M, Bidossi A, Logoluso N, Pellegrini A, De Vecchi E. A spacer infection by Candida albicans secondary to a Staphylococcus capitis prosthetic joint infection: a case report. *BMC Infectious Diseases.* 2021/05/04 2021;21(1):416.

13.	Brooks DH, Pupparo F. Successful salvage of a primary total knee arthroplasty infected with Candida parapsilosis. *The Journal of Arthroplasty.* 1998/09/01/ 1998;13(6):707–712.

14.	Bruce ASW, Kerry RM, Norman P, Stockley I. Fluconazole-impregnated beads in the management of fungal infection of prosthetic joints. *The Journal of Bone & Joint Surgery British Volume.* 2001;83-B(2):183–184.

15.	Burgo FJ, Mengelle DE, Abraham A, Kremer G, Autorino CM. Periprosthetic fungal infection of a hip caused by Trichosporon inkin. *Arthroplast Today.* Mar 2018;4(1):24–26.

16.	Carrega G, Cavagnaro L, Basso M, et al. Azole-resistant Candida albicans prosthetic joint infection treated with prolonged administration of anidulafungin and two-stage exchange with implant of a mega-prosthesis. *J Chemother.* Dec 2017;29(6):386–388.

17.	Wang-Kei C, Kwong-Yin C, Kin-Wing C, Kwok-Hing C. Candida Parapsilosis Total Hip Arthroplasty Infection: Case Report and Literature Review. *Journal of Orthopaedics, Trauma and Rehabilitation.* 2013/06/01 2013;17(1):33–36.

18.	Chong YC, Tse TST. A case of candida parapsilosis periprosthetic joint infection: Case report and literature review. *Journal of Orthopaedics, Trauma and Rehabilitation.* 2022/06/01 2022;29(1):22104917221075826.

19.	Çifci Ş, Aktuğ Demir N, Sümer Ş, Ural O, Çölkesen F. A Case of Prosthetic Infection with Candida Growth in the Knee Joınt. *Genel Tıp Dergisi.* April 2023;33(2):239–241.

20.	Ciochetto Z, Georgen M, Hadro A, et al. Reactivation of Coccidioides immitis in a Prosthetic Knee after Initiation of Chemotherapy. *Case Reports in Hematology.* 2021/01/01 2021;2021(1):3964465.

21.	Cobo F, Rodríguez-Granger J, López EM, et al. Candida-induced prosthetic joint infection. A literature review including 72 cases and a case report. *Infectious Diseases.* 2017/02/01 2017;49(2):81–94.

22.	Cs N, Mammu S, Unnikrishnan V, Sulaiman S. Delayed Mycotic Prosthetic Joint Infection: A Rare Case Report. *J Orthop Case Rep.* Feb 2025;15(2):155–160.

23.	Cushing RD, Fulgenzi WR. Synovial fluid levels of fluconazole in a patient with Candida parapsilosis prosthetic joint infection who had an excellent clinical response. *The Journal of Arthroplasty.* 1997/12/01/ 1997;12(8):950.

24.	Darouiche RO, Hamill RJ, Musher DM, Young EJ, Harris RL. Periprosthetic Candidal Infections Following Arthroplasty. *Reviews of Infectious Diseases.* 1989;11(1):89–96.

25.	Deelstra JJ, Neut D, Jutte PC. Successful Treatment of Candida Albicans–Infected Total Hip Prosthesis With Staged Procedure Using an Antifungal-Loaded Cement Spacer. *The Journal of Arthroplasty.* 2013/02/01/ 2013;28(2):374.e375–374.e378.

26.	DeHart DJ. Use of Itraconazole for Treatment of Sporotrichosis Involving a Knee Prosthesis. *Clinical Infectious Diseases.* 1995;21(2):450–450.

27.	Dumaine V, Eyrolle L, Baixench MT, et al. Successful treatment of prosthetic knee Candida glabrata infection with caspofungin combined with flucytosine. *International Journal of Antimicrobial Agents.* 2008/04/01/ 2008;31(4):398–399.

28.	Dunkley AB, Leslie IJ. Candida Infection of a Silicone Metacarpophalangeal Arthroplasty. *Journal of Hand Surgery.* 1997/06/01 1997;22(3):423–424.

29.	Dutronc H, Dauchy FA, Cazanave C, et al. Candida prosthetic infections: Case series and literature review. *Scandinavian Journal of Infectious Diseases.* 2010/12/01 2010;42(11-12):890–895.

30.	Evans RP, Nelson CL. Staged reimplantation of a total hip prosthesis after infection with Candida albicans. A report of two cases. *JBJS.* 1990;72(10).

31.	Fabry K, Verheyden F, Nelen G. Infection of a total knee prosthesis by Candida glabrata: a case report. *Acta Orthop Belg.* Feb 2005;71(1):119–121.

32.	Fowler VG, Jr., Nacinovich FM, Alspaugh JA, Corey GR. Prosthetic Joint Infection Due to Histoplasma capsulatum: Case Report and Review. *Clinical Infectious Diseases.* 1998;26(4):1017–1017.

33.	Frank FA, Krampitz B, Steiner J, et al. Evaluation and testing of polymethylmetacrylic (PMMA) bone cements with admixed Amphotericin B. *Journal of Orthopaedic Surgery and Research.* 2025/02/08 2025;20(1):151.

34.	Frieler S, Yilmaz E, Goodmanson R, Hanusrichter Y, Schildhauer TA, Baecker H. Conversion From Knee Arthrodesis Back to Arthroplasty: A Particular Challenge in Combination With Fungal Periprosthetic Joint Infection. *Arthroplast Today.* Dec 2020;6(4):1038–1044.

35.	Fukasawa N, Shirakura K. Candida arthritis after total knee arthroplasty--a case of successful treatment without prosthesis removal. *Acta Orthop Scand.* Jun 1997;68(3):306–307.

36.	Gabriel S, Boin MA. Cryptococcus neoformans Periprosthetic Joint Infection in the Setting of Revision Shoulder Arthroplasty: A Case Report. *Cureus.* Oct 2024;16(10):e71253.

37.	Gautam VK, Mahajan S, Tiwari P. Rare case of prosthetic and non-prosthetic Candida arthritis of bilateral knee joints by Candida parapsilosis successfully treated with fluconazole: A diagnostic paradox. *IDCases.* 2025/01/01/ 2025;40:e02243.

38.	George JC, Chittaranjan BS, Babu S, George K. Candida parapsilosis in Late Prosthetic Joint Infections Among Immunocompetent Patients: A Case Series. *J Orthop Case Rep.* Aug 2024;14(8):185–191.

39.	George JC, Sainulabdeen J, Chittaranjan S, George K, Babu S. Single-Stage Revision With Fluconazole Monotherapy in Fungal Prosthetic Knee Joint Infections. *Arthroplast Today.* Feb 2021;7:238–241.

40.	Giordani FA, Kiernan B, Giordani M, Darrow M, Thorpe S, Meehan JP. Coccidioidomycosis in Joint Replacement: A Review of the Literature With Case Presentations. *Arthroplast Today.* Jun 2023;21:101123.

41.	Giovanoulis V, Vasiliadis AV, Koutserimpas C, et al. Prosthetic Knee Joint Infection Due to Candida lusitaniae: A Diagnostic and Therapeutic Odyssey: A Case Study. *Diagnostics.* 2022;12(11):2640.

42.	Goodman JS, Seibert DG, Reahl GE, Jr., Geckler RW. Fungal infection of prosthetic joints: a report of two cases. *J Rheumatol.* Jun 1983;10(3):494–495.

43.	Gottesman-Yekutieli T, Dan M, Shwartz O, Edelman A, Hendel D. Pseudallescheria boydii Infection of a Prosthetic Hip Joint—An Uncommon Infection in a Rare Location. *The American Journal of the Medical Sciences.* 2011/09/01/ 2011;342(3):250–253.

44.	Graw B, Woolson S, Huddleston JI. Candida Infection in Total Knee Arthroplasty with Successful Reimplantation. *J Knee Surg.* 2011/01/05 2010;23(03):169–174.

45.	Hall RL, Frost RM, Vasukutty NL, Minhas H. &lt;em&gt;Candida glabrata&lt;/em&gt;: an unusual fungal infection following a total hip replacement. *BMJ Case Reports.* 2012;2012:bcr–2012–006491.

46.	Halstead FD, Yates E, Onwukwe C, et al. An unwanted souvenir: Case report of delayed diagnosis of a coccidioidomycosis prosthetic joint infection. *Clinical Infection in Practice.* 2025/11/01/ 2025;28:100509.

47.	Hekman L, Yazdi N, Seu M, et al. A Candida Glabrata-Associated Prosthetic Joint Infection: Case Report and Literature Review. *Fed Pract.* Mar 2025;42(3):134–137.

48.	Henriquez AR, Crook B, Ruderman LV, Lorentz S, Klifto CS, Anakwenze O. Concomitant joint infection in shoulder periprosthetic fracture patients: a case series. *Seminars in Arthroplasty: JSES.* 2025;35(3):448–453.

49.	Hirsch BE, Farber BF, Shapiro JF, Kennelly S. Successful Treatment of Aureobasidium pullulans Prosthetic Hip Infection. *Infectious Diseases in Clinical Practice.* 1996;5(3).

50.	Hnatow M, Martin BC, Herrera E. Recalcitrant Fungal Prosthetic Joint Infection of the Knee Cleared Using Voriconazole via Intra-Articular Catheters. *Clinical Case Reports.* 2025/10/01 2025;13(10):e71257.

51.	Ishak N, Pachayappan K, Hwa CL, Mohamad Kamil MA. A case of prosthetic joint infection due to opportunist yeast, Cyberlindnera fabianii. *Current Medical Mycology.* 2023;9(3):45–49.

52.	Jenny JY, Goukodadja O, Boeri C, Gaudias J. May one-stage exchange for Candida albicans peri-prosthetic infection be successful? *Orthopaedics & Traumatology: Surgery & Research.* 2016/02/01/ 2016;102(1):127–129.

53.	Johannsson B, Callaghan JJ. Prosthetic hip infection due to Cryptococcus neoformans: case report. *Diagnostic Microbiology and Infectious Disease.* 2009/05/01/ 2009;64(1):76–79.

54.	Keck JM, Dare RK, Jenkins MB, et al. It’s Here, It’s There, There’s Fungi Everywhere: A Case Series Utilizing Rezafungin for Invasive Candidiasis. *Infectious Diseases and Therapy.* 2025/04/01 2025;14(4):889–895.

55.	Keuning MC, Al Moujahid A, Zijlstra WP. Prosthetic Joint Infection of a Revision Knee Arthroplasty with Candida parapsilosis. *Case Reports in Orthopedics.* 2019/01/01 2019;2019(1):3634519.

56.	Kobayashi T, Lawler E, Samra H, Ford B, Sekar P. Prosthetic Finger Joint Infection Due to Aspergillus terreus. *Open Forum Infectious Diseases.* 2021;8(1):ofaa614.

57.	Koutserimpas C, Naoum S, Alpantaki K, et al. Fungal Prosthetic Joint Infection in Revised Knee Arthroplasty: An Orthopaedic Surgeon’s Nightmare. *Diagnostics.* 2022;12(7):1606.

58.	Koutserimpas C, Samonis G, Velivassakis E, Iliopoulou-Kosmadaki S, Kontakis G, Kofteridis DP. Candida glabrata prosthetic joint infection, successfully treated with anidulafungin: A case report and review of the literature. *Mycoses.* 2018/04/01 2018;61(4):266–269.

59.	Kuner E, Jöckel JA, Orler R, Nüesch R. Periprosthetic knee infection by Mycobacterium bovis and Candida guilliermondii in the context of a zoonosis: a case report and review of the literature. *Journal of Medical Case Reports.* 2019/03/08 2019;13(1):54.

60.	Kurmis AP. Eradicating Fungal Periprosthetic TKA "Super-infection": Review of the Contemporary Literature and Consideration of Antibiotic-Impregnated Dissolving Calcium Sulfate Beads as a Novel PJI Treatment Adjunct. *Arthroplast Today.* Apr 2021;8:163–170.

61.	Kuthan R, Zaremba-Wróblewski GL, Ott F, Soltaninia D. Septic Obturation of a Knee Endoprosthesis Caused by Aspergillus clavatus. *Pathogens.* 2023;12(10):1270.

62.	Lackner M, De Man FH, Eygendaal D, et al. Severe prosthetic joint infection in an immunocompetent male patient due to a therapy refractory Pseudallescheria apiosperma. *Mycoses.* 2011/10/01 2011;54(s3):22–27.

63.	Lazzarini L, Manfrin V, de Lalla F. Candidal prosthetic hip infection in a patient with previous candidal septic arthritis. *The Journal of Arthroplasty.* 2004/02/01/ 2004;19(2):248–252.

64.	Lejko-Zupanc T, Možina E, Vrevc F. Caspofungin as treatment for Candida glabrata hip infection. *International Journal of Antimicrobial Agents.* 2005/03/01/ 2005;25(3):273–274.

65.	Lerch K, Kalteis T, Schubert T, Lehn N, Grifka J. Prosthetic joint infections with osteomyelitis due to Candida albicans. *Mycoses.* 2003/12/01 2003;46(11-12):462–466.

66.	Leylabadlo HE, Zeinalzadeh E, Akbari NA, Kafil HS. Malassezia species infection of the synovium after total knee arthroplasty surgery. *GMS Hyg Infect Control.* 2016;11:Doc19.

67.	Li M, Fan M, Zhang Y, Xiao J, Liu T, Yu Q. Efficacy of DAIR in managing Candida parapsilosis-infected total knee arthroplasty with five-year follow-up: A case report and review of literature. *Medicine.* 2023;102(47).

68.	Lidder S, Tasleem A, Masterson S, Carrington RWJ. &lt;em&gt;Candida tropicalis&lt;/em&gt;: diagnostic dilemmas for an unusual prosthetic hip infection. *Journal of the Royal Army Medical Corps.* 2013;159(2):123.

69.	Lim EV, Stern PJ. Candida infection after implant arthroplasty. A case report. *JBJS.* 1986;68(1).

70.	Lin Y-J, Su T, Yang L, Chen G-X. Mixed bacterial-fungal infection following total hip arthroplasty: A case report. *Chinese Journal of Traumatology.* 2022/01/01/ 2022;25(1):32–36.

71.	Liu Y, Lin J. Periprosthetic bacterial and fugal infection after total knee arthroplasty with one-stage debridement: a case report. *Journal of Medical Case Reports.* 2024/04/13 2024;18(1):177.

72.	Longfellow GA, Himmelberg SM, Jeffs AD, Draeger RW. Fusarium Species Fungal Prosthetic Joint Infection of the Proximal Interphalangeal Joint After Silicone Arthroplasty: A Case Report. *Clin Med Insights Case Rep.* 2025;18:11795476251377947.

73.	MacLean IS, Day SR, Moore CC, Browne JA. Blastomycosis infection of the knee treated with staged total knee arthroplasty. *The Knee.* 2015/12/01/ 2015;22(6):669–671.

74.	Mafrachi BW, Debei AHA, Muhtaseb FMA, Al-Ajlouni JM, Hammad YS. Fungal Prosthetic Joint Infection Following Total Knee Arthroplasty: A Case Report. *J Orthop Case Rep.* Feb 2021;11(2):95–98.

75.	Marcomini MP, Iqbal J, Bennett D. Rare Pseudotumor in Ceramic-On-Ceramic Total Hip Replacement with Concomitant Fungal Periprosthetic Joint Infection: A Case Report. *Am J Case Rep.* Sep 6 2023;24:e941164.

76.	Meiyappan A, Villa JM, Sabesan VJ, Patel PD, Suarez JC. Histoplasma capsulatum periprosthetic knee infection complicated by autoimmune-mediated systemic inflammatory response syndrome. *Arthroplast Today.* Jun 2019;5(2):135–138.

77.	Mello López A, da Silva Neto LA, de Oliveira VF, de Carvalho VC, de Oliveira PRD, Lima ALLM. Orthopedic infections due to Trichosporon species: Case series and literature review. *Medical Mycology.* 2023;61(1):myad001.

78.	Merrer J, Dupont B, Nieszkowska A, De Jonghe B, Outin H. Candida albicans Prosthetic Arthritis Treated with Fluconazole Alone. *Journal of Infection.* 2001/05/01/ 2001;42(3):208–209.

79.	Morimoto Y, Yo H, Ohashi H. Two-stage revision using antifungal-loaded cement beads for the treatment of Candida infection following revision total hip arthroplasty: A case report. *J Orthop Sci.* May 2021;26(3):505–509.

80.	Murata Y, Okabe S, Tamagawa S, Ohnishi H, Sakai A. Candidal Prosthetic Hip Infection in a Patient with Previous Candidal Sepsis - A Case Report. *J uoeh.* 2020;42(4):327–330.

81.	Nadarevic E, McHugh J, Vergidis P. Candida glabrata Prosthetic Joint Infection Managed with Ibrexafungerp. *J Fungi (Basel).* Jun 11 2025;11(6).

82.	Nowbakht C, Garrity K, Webber N, Eraso J, Ostrosky-Zeichner L. Prosthetic Joint Infection Due to Histoplasma capsulatum Complicating a Total Knee Arthroplasty. *Open Forum Infect Dis.* Summer 2017;4(3):ofx118.

83.	Oenning S, Moellenbeck B, Gosheger G, et al. Fungal Periprosthetic Knee Joint Infection in a Patient with Metamizole-Induced Agranulocytosis. *Arthroplast Today.* Dec 2020;6(4):726–730.

84.	Ornell SS, Dang KH, Bois AJ, Dutta AK. Fungal Infection following Total Elbow Arthroplasty. *Case Rep Orthop.* 2019;2019:7927914.

85.	Pasticci MB, Papalini C, Leli A, Bruno G. Two-stage revision and systemic antifungal therapy of Candida glabrata primary prosthetic hip infection successfully treated: a case report. *J Med Case Rep.* May 21 2019;13(1):151.

86.	Patel M, Kodra J, Keener K, Singh R, Jha P. Candida Auris Total Knee Arthroplasty Infection in an Immunocompetent Individual: Case Report and Literature Review. *Wmj.* 2025;124(3):291–295.

87.	Pozzi L, Schläppi M, Livio F, Blatter S, Achermann Y, Wahl P. Joint fluid concentrations of amphotericin B after local application with calcium sulphate-report of 2 cases. *Apmis.* Nov 2023;131(11):567–573.

88.	Presman B, Christensen JJ, Broeng L, Marckmann P, Nielsen XC. Deep Infection With Candida albicans Following Total Hip Replacement: Two Case Reports. *Infectious Diseases in Clinical Practice.* 2014;22(4).

89.	Radha S, Siddiqui BM, Senevirathna S, Harrison JW. Candida albicans infection of a reverse-polarity shoulder replacement in a patient with rheumatoid arthritis treated with etanercept. *J Clin Rheumatol.* Sep 2012;18(6):323.

90.	Rajme-López S, González-Lara MF, Rangel-Cordero A, Ponce-de-León A. Histoplasma capsulatum prosthetic joint infection. *Med Mycol Case Rep.* Jun 2023;40:33–35.

91.	Ramamohan N, Zeineh N, Grigoris P, Butcher I. Candida glabrata infection after total hip arthroplasty. *J Infect.* Jan 2001;42(1):74–76.

92.	Reddy KJ, Shah JD, Kale RV, Reddy TJ. Fungal prosthetic joint infection after total knee arthroplasty. *Indian J Orthop.* Sep 2013;47(5):526–529.

93.	Sarzaeem MM, Beigi AN, Darestani RT, Omrani FA, Baroutkoub M, Rasi AM. Candida albicans Prosthetic Joint Infection After Total Knee Arthroplasty: A Rare Case Report. *Adv Biomed Res.* 2022;11:111.

94.	Sebastian S, Malhotra R, Pande A, Gautam D, Xess I, Dhawan B. Staged Reimplantation of a Total Hip Prosthesis After Co-infection with Candida tropicalis and Staphylococcus haemolyticus: A Case Report. *Mycopathologia.* Jun 2018;183(3):579–584.

95.	Selmon GP, Slater RN, Shepperd JA, Wright EP. Successful 1-stage exchange total knee arthroplasty for fungal infection. *J Arthroplasty.* Jan 1998;13(1):114–115.

96.	Shah NB, Shoham S, Nayak S. Cryptococcus neoformans prosthetic joint infection: case report and review of the literature. *Mycopathologia.* Apr 2015;179(3-4):275–278.

97.	Simonian PT, Brause BD, Wickiewicz TL. Candida infection after total knee arthroplasty. Management without resection or amphotericin B. *J Arthroplasty.* Oct 1997;12(7):825–829.

98.	Skedros JG, Keenan KE, Updike WS, Oliver MR. Failed Reverse Total Shoulder Arthroplasty Caused by Recurrent Candida glabrata Infection with Prior Serratia marcescens Coinfection. *Case Rep Infect Dis.* 2014;2014:142428.

99.	Song KY, Park C, Byun JH, et al. Fungal arthritis with adjacent osteomyelitis caused by Candida pelliculosa: a case report. *BMC Infect Dis.* Jun 22 2020;20(1):438.

100.	Springer J, Chatterjee S. Candida albicans prosthetic shoulder joint infection in a patient with rheumatoid arthritis on multidrug therapy. *J Clin Rheumatol.* Jan 2012;18(1):52–53.

101.	Tabliago NRA, Maish D, Martinez RM, Shelly MA, Yablonski B, Kwon T. Acremonium (Sarocladium) periprosthetic joint infection: case report, literature review, and proposed antifungal regimen. *BMC Infect Dis.* Dec 29 2022;22(1):964.

102.	Sharma A, Thakur K, Sharma A, Prasad M. Dual Organism Periprosthetic Joint Infection Following Total Knee Arthroplasty: A Case Report and One Year Follow-Up. *Journal of Arthroscopy and Joint Surgery.* 2024;11(1).

103.	Tiwari V, Shendre P, Nag S, Mishra M, Roy M, Dwidmuthe S. Peri-prosthetic Joint Infection due to Candida albicans in a Young Immunocompetent Patient - A Rare Case Report. *J Orthop Case Rep.* Feb 2024;14(2):160–164.

104.	Tummala SV, Lin E, Mujahed T, Beauchamp CP, Blair JE, Goulding KA. Rare Bipolaris Species Fungal Periprosthetic Hip Infection in an Immunocompetent Host: A Case Report. *JBJS Case Connect.* Oct 1 2023;13(4).

105.	Tunkel AR, Thomas CY, Wispelwey B. Candida prosthetic arthritis: report of a case treated with fluconazole and review of the literature. *Am J Med.* Jan 1993;94(1):100–103.

106.	Unfried RI, Krause LMF, Cezimbra HM, Pacheco LS, Larangeira JA, Ribeiro TA. A Retrospective Observational Cohort Study of Periprosthetic Hip Infection Treated by one-stage Method Including Cases With Bone Graft Reconstruction. *Clin Med Insights Arthritis Musculoskelet Disord.* 2022;15:11795441221090344.

107.	Vergison L, Schepens A, Liekens K, De Kesel R, Van der Bracht H, Victor J. Periprosthetic joint infection of a total hip arthroplasty with Candida parapsilosis. *Int J Surg Case Rep.* 2020;69:72–75.

108.	Wada M, Baba H, Imura S. Prosthetic knee Candida parapsilosis infection. *J Arthroplasty.* Jun 1998;13(4):479–482.

109.	White A, Goetz MB. Candida parapsilosis prosthetic joint infection unresponsive to treatment with fluconazole. *Clin Infect Dis.* Apr 1995;20(4):1068–1069.

110.	Williams BT, Hogan C, Damioli L. Two-Stage Revision Total Knee Arthroplasty for Chronic Histoplasma Capsulatum Prosthetic Joint Infection: A Case Report. *JBJS Case Connect.* Jul 1 2022;12(3).

111.	Wiwattanawarang N. Fungal periprosthetic joint infection after total knee arthroplasty. *J Med Assoc Thai.* Dec 2014;97(12):1358–1363.

112.	Wu CY, Huang HK, Wu PK, Chen WM, Lai MC, Chung LH. Acremonium species combined with Penicillium species infection in hip hemiarthroplasty: a case report and literature review. *Hip Int.* Dec 5 2014;24(6):656–659.

113.	Wu MH, Hsu KY. Candidal arthritis in revision knee arthroplasty successfully treated with sequential parenteral-oral fluconazole and amphotericin B-loaded cement spacer. *Knee Surg Sports Traumatol Arthrosc.* Feb 2011;19(2):273–276.

114.	Xiang Y, Xuan YY, Li G. Successful treatment for acute prosthetic joint infection due to MRSA and Candida albicans: a case report and literature review. *Ther Clin Risk Manag.* 2018;14:1133–1139.

115.	Xin J, Guo QS, Zhang HY, et al. Candidal periprosthetic joint infection after primary total knee arthroplasty combined with ipsilateral intertrochanteric fracture: A case report. *World J Clin Cases.* Nov 6 2020;8(21):5401–5408.

116.	Yang SH, Pao JL, Hang YS. Staged reimplantation of total knee arthroplasty after Candida infection. *J Arthroplasty.* Jun 2001;16(4):529–532.

117.	Younkin S, Evarts CM, Steigbigel RT. Candida parapsilosis infection of a total hip-joint replacement: successful reimplantation after treatment with amphotericin B and 5-fluorocytosine. A case report. *J Bone Joint Surg Am.* Jan 1984;66(1):142–143.

118.	Zuo Q, Dong L, Mu W, Zhou L, Hu T, Zhang H. Trichosporon asahii infection after total knee arthroplasty: A case report and review of the literature. *Can J Infect Dis Med Microbiol.* Jan–Feb 2015;26(1):47–51.

119.	Anagnostakos K, Grzega C, Sahan I, Geipel U, Becker SL. Occurrence of Rare Pathogens at the Site of Periprosthetic Hip and Knee Joint Infections: A Retrospective, Single-Center Study. *Antibiotics.* 2021;10(7):882.

120.	Anagnostakos K, Kelm J, Schmitt E, Jung J. Fungal Periprosthetic Hip and Knee Joint Infections: Clinical Experience With a 2-Stage Treatment Protocol. *The Journal of Arthroplasty.* 2012/02/01/ 2012;27(2):293–298.

121.	Azzam K, Parvizi J, Jungkind D, et al. Microbiological, Clinical, and Surgical Features of Fungal Prosthetic Joint Infections: A Multi-Institutional Experience. *JBJS.* 2009;91(Supplement_6).

122.	Baecker H, Frieler S, Geßmann J, Pauly S, Schildhauer TA, Hanusrichter Y. Three-stage revision arthroplasty for the treatment of fungal periprosthetic joint infection: outcome analysis of a novel treatment algorithm : a prospective study. *Bone Jt Open.* Aug 2021;2(8):671–678.

123.	Brown TS, Petis SM, Osmon DR, et al. Periprosthetic Joint Infection With Fungal Pathogens. *The Journal of Arthroplasty.* 2018/08/01/ 2018;33(8):2605–2612.

124.	Burr RG, Eikani CK, Adams WH, Hopkinson WJ, Brown NM. Predictors of Success With Chronic Antibiotic Suppression for Prosthetic Joint Infections. *The Journal of Arthroplasty.* 2022/08/01/ 2022;37(8, Supplement):S983–S988.

125.	Dinh A, McNally M, D’Anglejan E, et al. Prosthetic Joint Infections due to Candida Species: A Multicenter International Study. *Clinical Infectious Diseases.* 2025;80(2):347–355.

126.	Eckers F, Laux CJ, Schaller S, Berli M, Achermann Y, Fucentese SF. Risk factor analysis for above-knee amputation in patients with periprosthetic joint infection of the knee: a case-control study. *BMC Musculoskelet Disord.* Oct 18 2021;22(1):884.

127.	Enz A, Mueller SC, Warnke P, Ellenrieder M, Mittelmeier W, Klinder A. Periprosthetic Fungal Infections in Severe Endoprosthetic Infections of the Hip and Knee Joint—A Retrospective Analysis of a Certified Arthroplasty Centre of Excellence. *Journal of Fungi.* 2021;7(6):404.

128.	Escolà-Vergé L, Rodríguez-Pardo D, Lora-Tamayo J, et al. Candida periprosthetic joint infection: A rare and difficult-to-treat infection. *Journal of Infection.* 2018/08/01/ 2018;77(2):151–157.

129.	Gao Z, Li X, Du Y, Peng Y, Wu W, Zhou Y. Success Rate of Fungal Peri-Prosthetic Joint Infection Treated by 2-Stage Revision and Potential Risk Factors of Treatment Failure: A Retrospective Study. *Med Sci Monit.* Aug 10 2018;24:5549–5557.

130.	Geng L, Xu M, Yu L, et al. Risk factors and the clinical and surgical features of fungal prosthetic joint infections: A retrospective analysis of eight cases. *Exp Ther Med.* 2016/08/01 2016;12(2):991–999.

131.	Gonzalez MR, Clunk MJ, Acosta JI, Bedi ADS, Karczewski D, Lozano-Calderón SA. High Rates of Treatment Failure and Amputation in Modular Endoprosthesis Prosthetic Joint Infections Caused by Fungal Infections With Candida. *Clinical Orthopaedics and Related Research®.* 2024;482(7).

132.	Hartman CW, Daubach EC, Richard BT, et al. Predictors of Reinfection in Prosthetic Joint Infections Following Two-Stage Reimplantation. *The Journal of Arthroplasty.* 2022/07/01/ 2022;37(7, Supplement):S674–S677.

133.	Herndon CL, Rowe TM, Metcalf RW, et al. Treatment Outcomes of Fungal Periprosthetic Joint Infection. *The Journal of Arthroplasty.* 2023/11/01/ 2023;38(11):2436–2440.e2431.

134.	Hwang BH, Yoon JY, Nam CH, et al. Fungal peri-prosthetic joint infection after primary total knee replacement. *The Journal of Bone & Joint Surgery British Volume.* 2012;94-B(5):656–659.

135.	Jeon YS, Kim MK, Kwon DG, et al. Palliative arthroscopic debridement with continuous irrigation for infected total knee arthroplasty in high mortality risk patients. *International Orthopaedics.* 2023/01/01 2023;47(1):175–186.

136.	Ji B, Li G, Zhang X, et al. Effective single-stage revision using intra-articular antibiotic infusion after multiple failed surgery for periprosthetic joint infection. *The Bone & Joint Journal.* 2022;104-B(7):867–874.

137.	Ji B, Wahafu T, Li G, et al. Single-stage treatment of chronically infected total hip arthroplasty with cementless reconstruction. *The Bone & Joint Journal.* 2019;101-B(4):396–402.

138.	Ji B, Zhang X, Xu B, Guo W, Mu W, Cao L. Single-Stage Revision for Chronic Fungal Periprosthetic Joint Infection: An Average of 5 Years of Follow-Up. *The Journal of Arthroplasty.* 2017/08/01/ 2017;32(8):2523–2530.

139.	Kanar M. KNEE ARTHRODESIS WITH COMPUTER-ASSISTED EXTERNAL FIXATOR SYSTEM AFTER PROSTHETIC JOINT INFECTION FOR ELDERLY POPULATION. *The Official Scientific Journal of Turkish Geriatrics Society.* 2023:60.

140.	Karczewski D, Ren Y, Andronic O, et al. Candida periprosthetic joint infections — risk factors and outcome between albicans and non-albicans strains. *International Orthopaedics.* 2022/03/01 2022;46(3):449–456.

141.	Kazimoglu C, Yalcin N, Onvural B, Akcay S, Agus H. Debridement, Antibiotics, Irrigation, and Retention (DAIR) of the Prosthesis after Hip Hemiarthroplasty Infections. Does it Work? *The International Journal of Artificial Organs.* 2015/08/01 2015;38(8):454–460.

142.	Kim J-K, Lee D-Y, Kang D-W, Ro D-H, Lee MC, Han H-S. Efficacy of antifungal-impregnated cement spacer against chronic fungal periprosthetic joint infections after total knee arthroplasty. *The Knee.* 2018/08/01/ 2018;25(4):631–637.

143.	Kim Y-H, Park J-W, Jang Y-S. Long-term result of a second or third two-stage revision total knee arthroplasty for infected total knee arthroplasty. *Arthroplasty.* 2021/01/12 2021;3(1):8.

144.	Klatte TO, Kendoff D, Kamath AF, et al. Single-stage revision for fungal peri-prosthetic joint infection. *The Bone & Joint Journal.* 2014;96-B(4):492–496.

145.	Kuo F-C, Goswami K, Shohat N, Blevins K, Rondon AJ, Parvizi J. Two-Stage Exchange Arthroplasty Is a Favorable Treatment Option Upon Diagnosis of a Fungal Periprosthetic Joint Infection. *The Journal of Arthroplasty.* 2018/11/01/ 2018;33(11):3555–3560.

146.	Lachman JR, Ramos JA, DeOrio JK, Easley ME, Nunley JA, Adams SB. Outcomes of Acute Hematogenous Periprosthetic Joint Infection in Total Ankle Arthroplasty Treated With Irrigation, Debridement, and Polyethylene Exchange. *Foot & Ankle International.* 2018/11/01 2018;39(11):1266–1271.

147.	Lee W-S, Park KK, Cho B-W, Park JY, Kim I, Kwon HM. Risk factors for early septic failure after two-stage exchange total knee arthroplasty for treatment of periprosthetic joint infection. *Journal of Orthopaedics and Traumatology.* 2024/02/12 2024;25(1):6.

148.	McCulloch RA, Palmer AJ, Donaldson J, Kendrick BJ, Miles J, Taylor A. The Outcomes of Hip and Knee Fungal Periprosthetic Joint Infections: A Retrospective Cohort Study. *The Journal of Arthroplasty.* 2023/10/01/ 2023;38(10):2183–2187.e2181.

149.	Miller AO, Gamaletsou MN, Henry MW, et al. Successful treatment of Candida osteoarticular infections with limited duration of antifungal therapy and orthopedic surgical intervention. *Infectious Diseases.* 2015/03/04 2015;47(3):144–149.

150.	Phelan DM, Osmon DR, Keating MR, Hanssen AD. Delayed reimplantation arthroplasty for candidal prosthetic joint infection: a report of 4 cases and review of the literature. *Clin Infect Dis.* Apr 1 2002;34(7):930–938.

151.	Saconi ES, de Carvalho VC, de Oliveira PRD, Lima A. Prosthetic joint infection due to Candida species: Case series and review of literature. *Medicine (Baltimore).* Apr 2020;99(15):e19735.

152.	Sidhu MS, Cooper G, Jenkins N, Jeys L, Parry M, Stevenson JD. Prosthetic fungal infections: poor prognosis with bacterial co-infection. *Bone Joint J.* May 2019;101-b(5):582–588.

153.	Starnes V, Duggan J, Hollingshead C. Fungal Prosthetic Joint Infection: A Case Series and Review of the Literature. *Infect Dis Ther.* May 2024;13(5):1051–1065.

154.	Theil C, Schmidt-Braekling T, Gosheger G, Idelevich EA, Moellenbeck B, Dieckmann R. Fungal prosthetic joint infection in total hip or knee arthroplasty. *The Bone & Joint Journal.* 2019;101-B(5):589–595.

155.	Ueng SW, Lee CY, Hu CC, Hsieh PH, Chang Y. What is the success of treatment of hip and knee candidal periprosthetic joint infection? *Clin Orthop Relat Res.* Sep 2013;471(9):3002–3009.

156.	Wang HY, Zhang Md R, Luo ZY, et al. One-Stage Arthroplasty or Revision for Seronegative Infections in Hip and Knee. *Orthop Surg.* Feb 2020;12(1):38–49.

157.	Wang QJ, Shen H, Zhang XL, et al. Staged reimplantation for the treatment of fungal peri-prosthetic joint infection following primary total knee arthroplasty. *Orthop Traumatol Surg Res.* Apr 2015;101(2):151–156.

158.	Whiteside LA, Roy ME. One-stage Revision With Catheter Infusion of Intraarticular Antibiotics Successfully Treats Infected THA. *Clin Orthop Relat Res.* Feb 2017;475(2):419–429.

159.	Xu J, Sun W, Wang Y, et al. Two-Stage Treatment Protocol of Fungal Periprosthetic Hip and Knee Joint Infections: the Clinical Experience from a Single Center Experience. *Acta Chir Orthop Traumatol Cech.* 2024;91(1):52–56.

160.	Yang HY, Shin HH, Kim JW, Seon JK. The fate of fungal periprosthetic joint infection after total knee arthroplasty. *Int Orthop.* Nov 2023;47(11):2727–2735.
